# Supplementary material for: Alpha-synuclein alters the faecal viromes of rats in a gut-initiated model of Parkinson’s disease
Source: Commun Biol. 2021 Sep 29;4:1140. doi: 10.1038/s42003-021-02666-1 (PMC8481466; doi:10.1038/s42003-021-02666-1)
Supplement: Supplementary file 1 — Supplementary material [file 42003_2021_2666_MOESM1_ESM.pdf]

Supplementary table 1. Alpha-diversity analysis of rat faecal viromes. Wilcoxon-paired statistical comparisons of rat faecal virome alpha-diversities, with Bonferroni correction, using both viral and WGS sequencing data.

Data	Group 1	Group 2	P−value	Bonferroni P−value
Virome	Sham	LPS	0.394	1.000
WGS	Sham	LPS	0.0177	0.270
Virome	Sham	Monomer	0.122	1.000
WGS	Sham	Monomer	0.4745	1.000
Virome	Sham	Monomer + LPS	0.083	1.000
WGS	Sham	Monomer + LPS	0.2447	1.000
Virome	Sham	PFF	0.062	0.920
WGS	Sham	PFF	0.0380	0.570
Virome	Sham	PFF + LPS	0.525	1.000
WGS	Sham	PFF + LPS	0.1837	1.000
Virome	LPS	Monomer	0.357	1.000
WGS	LPS	Monomer	0.0145	0.220
Virome	LPS	Monomer + LPS	0.083	1.000
WGS	LPS	Monomer + LPS	0.3687	1.000
Virome	LPS	PFF	0.219	1.000
WGS	LPS	PFF	0.6598	1.000
Virome	LPS	PFF + LPS	0.782	1.000
WGS	LPS	PFF + LPS	0.3514	1.000
Virome	Monomer	Monomer + LPS	0.483	1.000
WGS	Monomer	Monomer + LPS	0.0773	1.000
Virome	Monomer	PFF	0.636	1.000
WGS	Monomer	PFF	0.0054	0.081
Virome	Monomer	PFF + LPS	0.386	1.000
WGS	Monomer	PFF + LPS	0.1434	1.000
Virome	Monomer + LPS	PFF	0.791	1.000
WGS	Monomer + LPS	PFF	0.1038	1.000
Virome	Monomer + LPS	PFF + LPS	0.097	1.000
WGS	Monomer + LPS	PFF + LPS	0.8391	1.000
Virome	PFF	PFF + LPS	0.192	1.000
WGS	PFF	PFF + LPS	0.1427	1.000

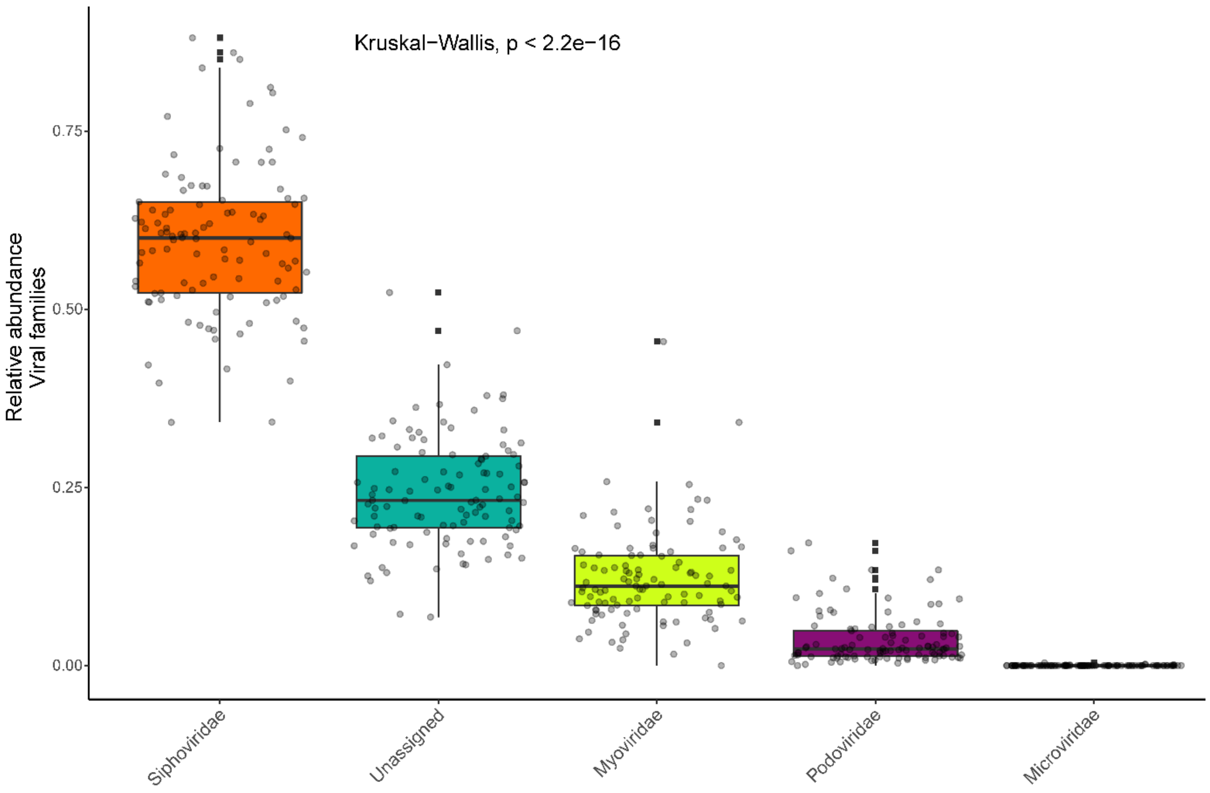

Supplementary figure 1. Relative abundance of predicted viral taxa in WGS data. Aggregation of sequencing data for each rat faecal sample investigated by Demovir predicted viral taxonomic assignment.

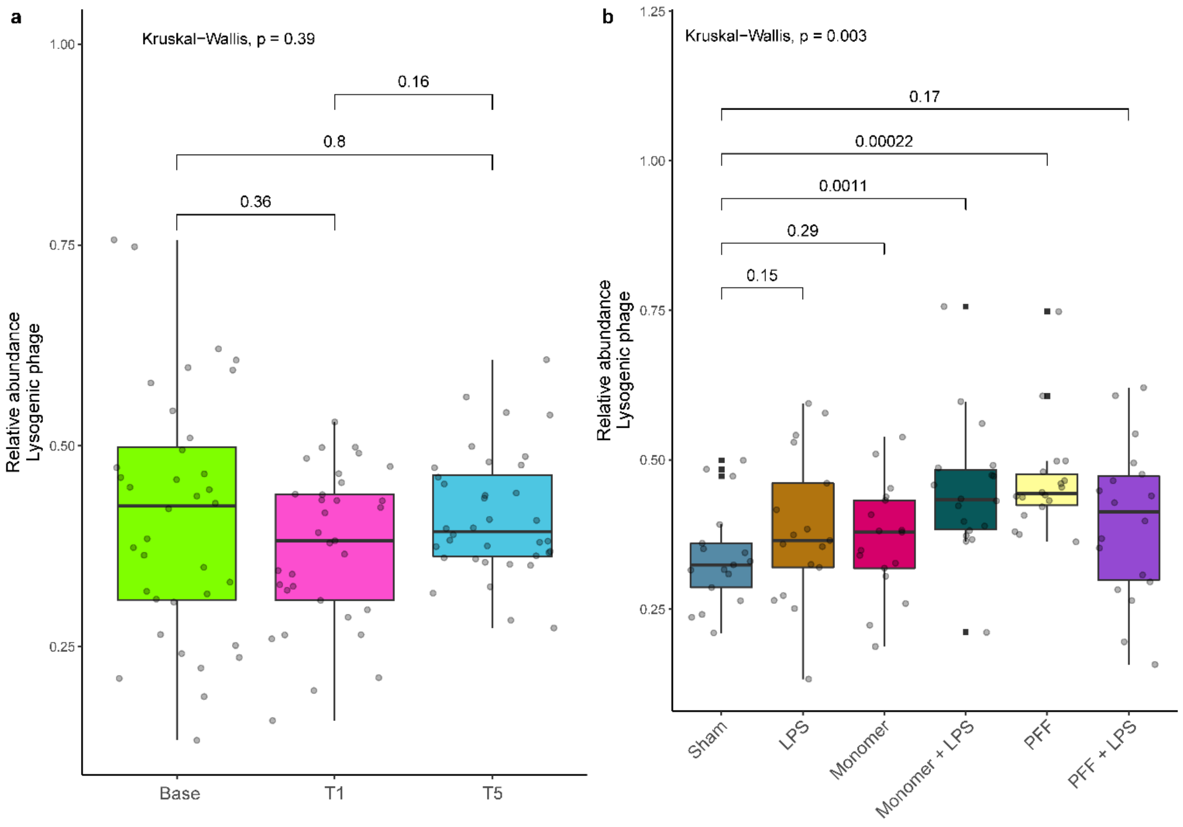

Supplementary figure 2. Changes in the relative abundance of rat faecal lysogenic phages (a) over time and (b) by treatment condition. P-values for specific group comparisons were performed using Wilcoxon-paired tests, while the Kruskal-Wallis test was performed across all groups.

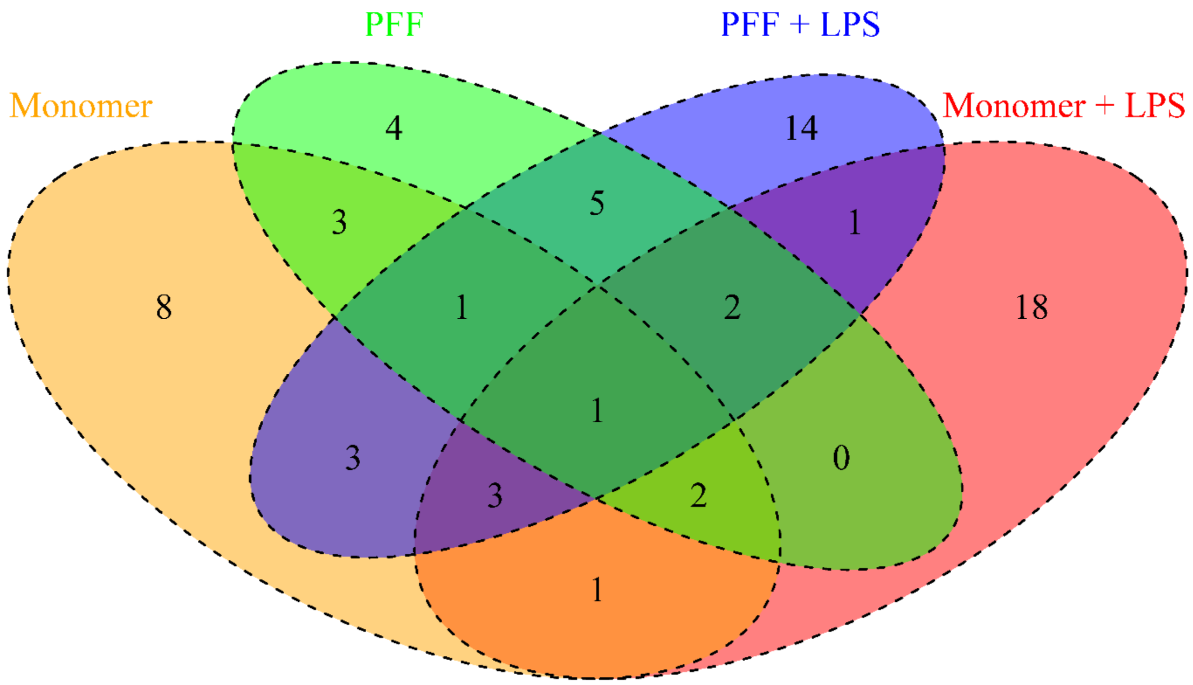

Supplementary figure 3. Overlapping rat faecal viruses differentially altered by α-synuclein treatment. Venn diagram indicating rat faecal viruses and viral clusters similarly altered across the α-syn monomer, α-syn monomer and LPS, α-syn PFF, and α-syn PFF and LPS, treatment groups.
