# Supplementary material for: Alpha-synuclein alters the faecal viromes of rats in a gut-initiated model of Parkinson’s disease
Source: Commun Biol. 2021 Sep 29;4:1140. doi: 10.1038/s42003-021-02666-1 (PMC8481466; doi:10.1038/s42003-021-02666-1)
Supplement: Supplementary file 2 — Description of Additional Supplementary Files [file 42003_2021_2666_MOESM2_ESM.pdf]

Description of Additional Supplementary Files

File name: Supplementary Data

Description: All processed sequencing output, R code, and metadata are provided as Supplementary Data and also available through FigShare with a link provided in main text.
