# Supplementary material for: Alpha-synuclein alters the faecal viromes of rats in a gut-initiated model of Parkinson’s disease
Source: Commun Biol. 2021 Sep 29;4:1140. doi: 10.1038/s42003-021-02666-1 (PMC8481466; doi:10.1038/s42003-021-02666-1)
Supplement: Supplementary file 3 — Supplementary data [file 42003_2021_2666_MOESM3_ESM.zip › Supp_data_RatPD_wDarkMatter/Output_images/Fig5.pdf]

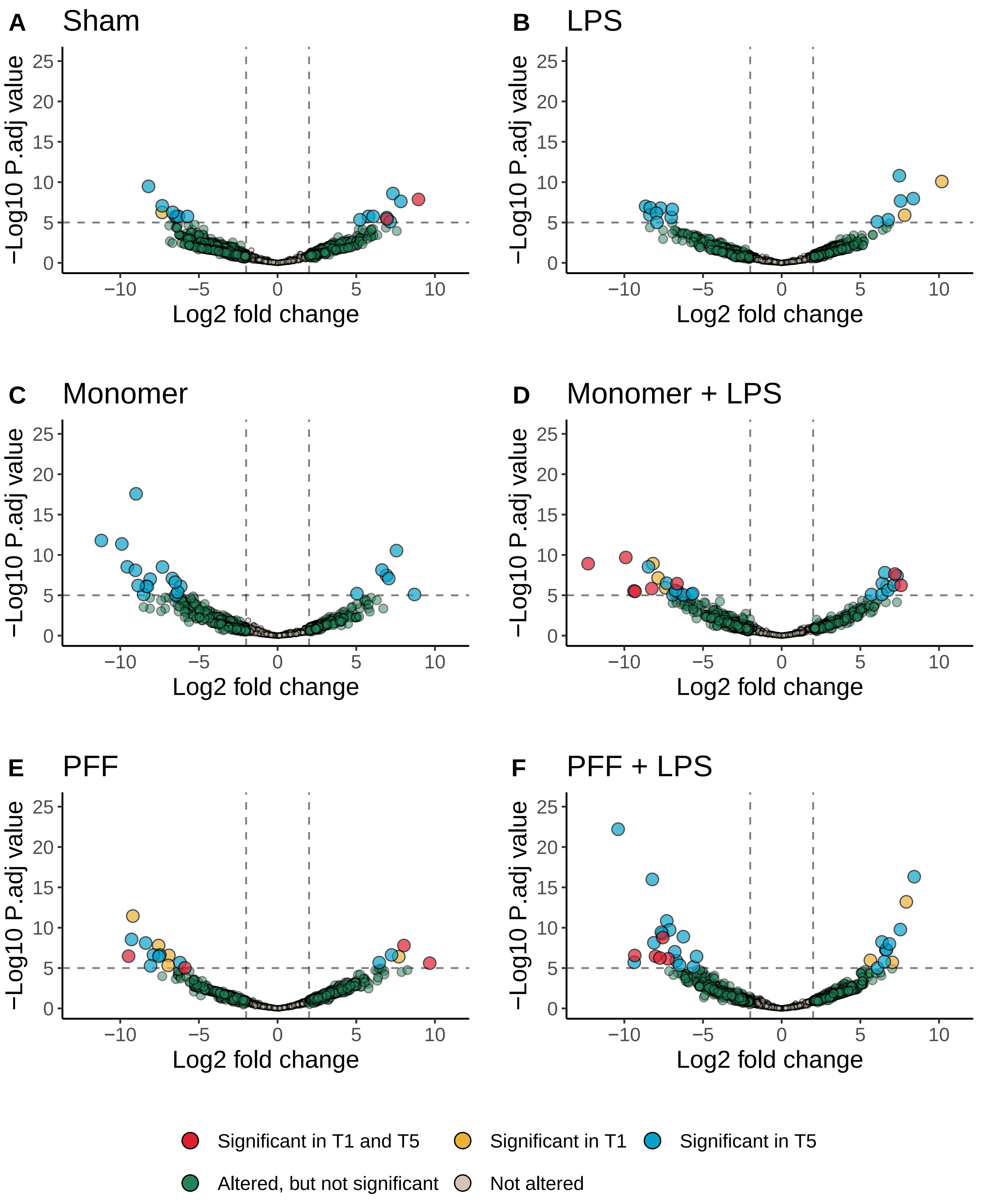
A
Sham
B
LPS
C
Monomer
D
Monomer + LPS
E
PFF
F
PFF + LPS
−Log10 P.adj value
Log2 fold change
0
5
10
15
20
25
−10
−5
Significant in T1 and T5
Significant in T1
Significant in T5
Altered, but not significant
Not altered
