# Supplementary material for: Alpha-synuclein alters the faecal viromes of rats in a gut-initiated model of Parkinson’s disease
Source: Commun Biol. 2021 Sep 29;4:1140. doi: 10.1038/s42003-021-02666-1 (PMC8481466; doi:10.1038/s42003-021-02666-1)
Supplement: Supplementary file 3 — Supplementary data [file 42003_2021_2666_MOESM3_ESM.zip › Supp_data_RatPD_wDarkMatter/Output_images/Supp_alpha_div.pdf]

| | Data | Group 1 | Group 2 | P−value | Bonferroni P−value | Significant |
|---|---|---|---|---|---|---|
| 1 | Virome | Sham | LPS | 0.394 | 1.000 | ns |
| 2 | WGS | Sham | LPS | 0.0177 | 0.270 | * |
| 3 | Virome | Sham | Monomer | 0.122 | 1.000 | ns |
| 4 | WGS | Sham | Monomer | 0.4745 | 1.000 | ns |
| 5 | Virome | Sham | Monomer + LPS | 0.083 | 1.000 | ns |
| 6 | WGS | Sham | Monomer + LPS | 0.2447 | 1.000 | ns |
| 7 | Virome | Sham | PFF | 0.062 | 0.920 | ns |
| 8 | WGS | Sham | PFF | 0.0380 | 0.570 | * |
| 9 | Virome | Sham | PFF + LPS | 0.525 | 1.000 | ns |
| 10 | WGS | Sham | PFF + LPS | 0.1837 | 1.000 | ns |
| 11 | Virome | LPS | Monomer | 0.357 | 1.000 | ns |
| 12 | WGS | LPS | Monomer | 0.0145 | 0.220 | * |
| 13 | Virome | LPS | Monomer + LPS | 0.083 | 1.000 | ns |
| 14 | WGS | LPS | Monomer + LPS | 0.3687 | 1.000 | ns |
| 15 | Virome | LPS | PFF | 0.219 | 1.000 | ns |
| 16 | WGS | LPS | PFF | 0.6598 | 1.000 | ns |
| 17 | Virome | LPS | PFF + LPS | 0.782 | 1.000 | ns |
| 18 | WGS | LPS | PFF + LPS | 0.3514 | 1.000 | ns |
| 19 | Virome | Monomer | Monomer + LPS | 0.483 | 1.000 | ns |
| 20 | WGS | Monomer | Monomer + LPS | 0.0773 | 1.000 | ns |
| 21 | Virome | Monomer | PFF | 0.636 | 1.000 | ns |
| 22 | WGS | Monomer | PFF | 0.0054 | 0.081 | ** |
| 23 | Virome | Monomer | PFF + LPS | 0.386 | 1.000 | ns |
| 24 | WGS | Monomer | PFF + LPS | 0.1434 | 1.000 | ns |
| 25 | Virome | Monomer + LPS | PFF | 0.791 | 1.000 | ns |
| 26 | WGS | Monomer + LPS | PFF | 0.1038 | 1.000 | ns |
| 27 | Virome | Monomer + LPS | PFF + LPS | 0.097 | 1.000 | ns |
| 28 | WGS | Monomer + LPS | PFF + LPS | 0.8391 | 1.000 | ns |
| 29 | Virome | PFF | PFF + LPS | 0.192 | 1.000 | ns |
| 30 | WGS | PFF | PFF + LPS | 0.1427 | 1.000 | ns |
