# Supplementary material for: Alpha-synuclein alters the faecal viromes of rats in a gut-initiated model of Parkinson’s disease
Source: Commun Biol. 2021 Sep 29;4:1140. doi: 10.1038/s42003-021-02666-1 (PMC8481466; doi:10.1038/s42003-021-02666-1)
Supplement: Supplementary file 3 — Supplementary data [file 42003_2021_2666_MOESM3_ESM.zip › Supp_data_RatPD_wDarkMatter/Output_images/Supp_lyso_treatments.pdf]

	Group 1	Group 2	P−value	Bonferroni P−value	Significant
1	Sham	LPS	0.14971	1.0000	ns
2	Sham	Monomer	0.28973	1.0000	ns
3	Sham	Monomer + LPS	0.00109	0.0160	**
4	Sham	PFF	0.00022	0.0033	***
5	Sham	PFF + LPS	0.17294	1.0000	ns
6	LPS	Monomer	0.73386	1.0000	ns
7	LPS	Monomer + LPS	0.07174	1.0000	ns
8	LPS	PFF	0.02454	0.3700	*
9	LPS	PFF + LPS	0.68366	1.0000	ns
10	Monomer	Monomer + LPS	0.02685	0.4000	*
11	Monomer	PFF	0.00382	0.0570	**
12	Monomer	PFF + LPS	0.44281	1.0000	ns
13	Monomer + LPS	PFF	0.56278	1.0000	ns
14	Monomer + LPS	PFF + LPS	0.33885	1.0000	ns
15	PFF	PFF + LPS	0.15164	1.0000	ns
