# Supplementary figures and images for: Alpha-synuclein alters the faecal viromes of rats in a gut-initiated model of Parkinson’s disease
Source: Commun Biol. 2021 Sep 29;4:1140. doi: 10.1038/s42003-021-02666-1 (PMC8481466; doi:10.1038/s42003-021-02666-1)

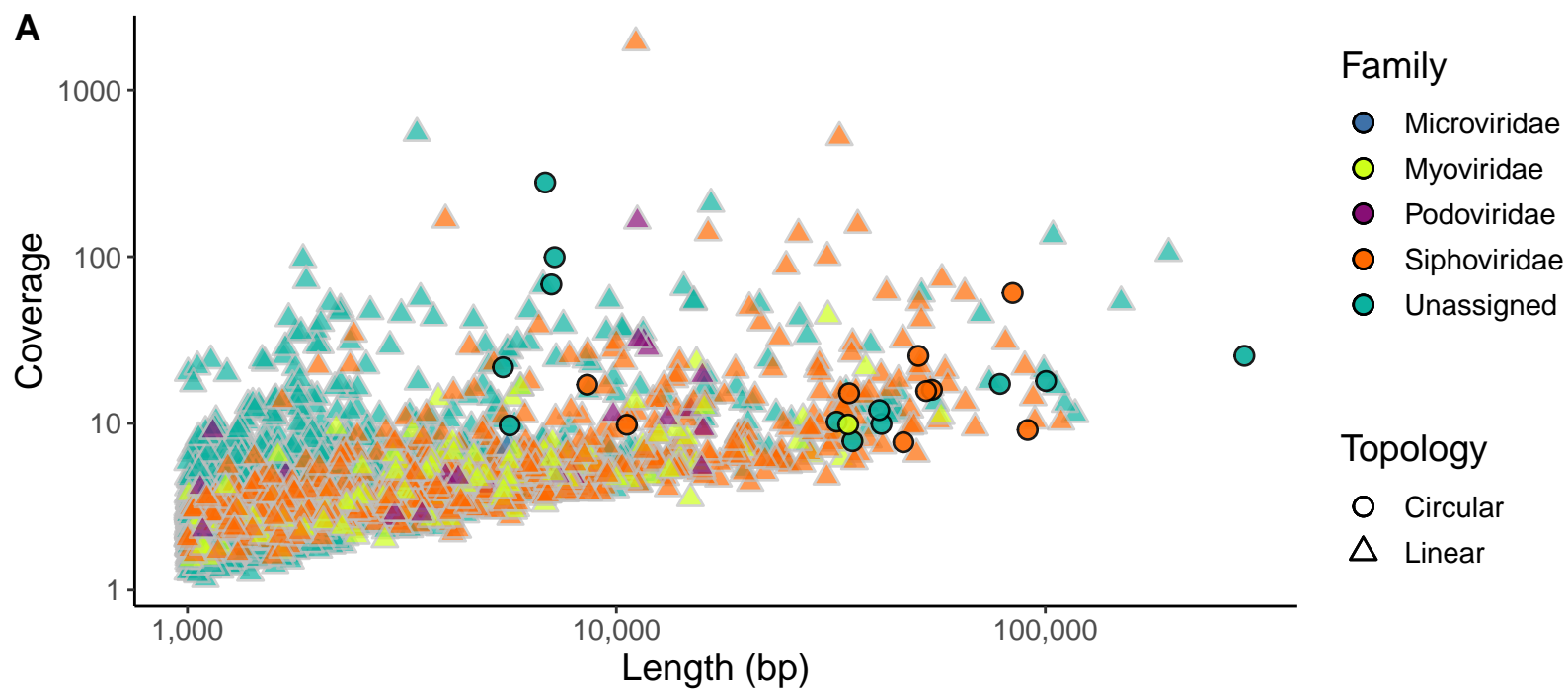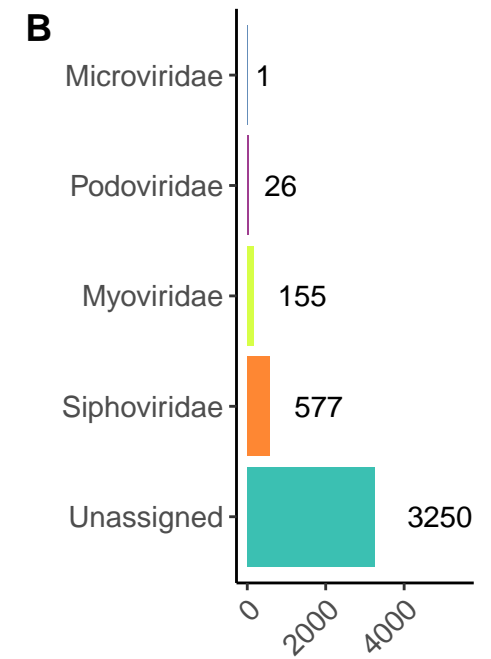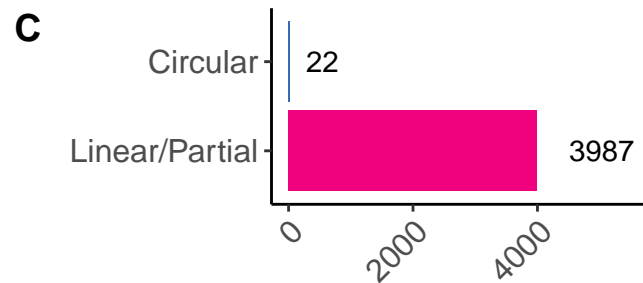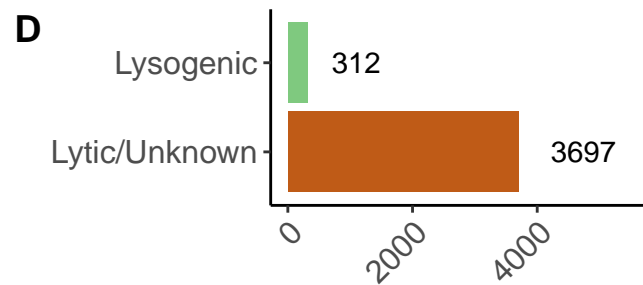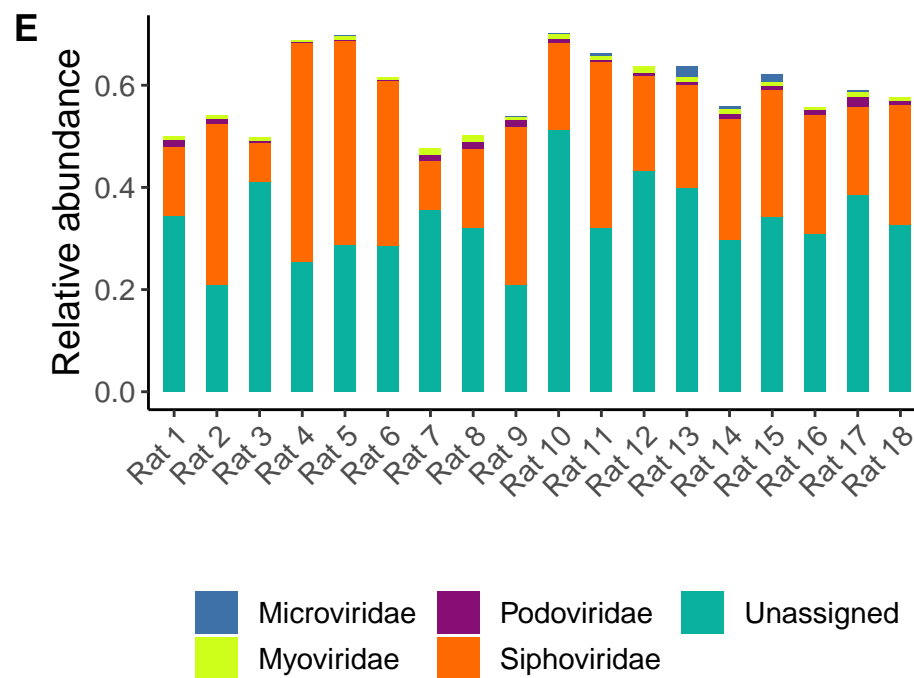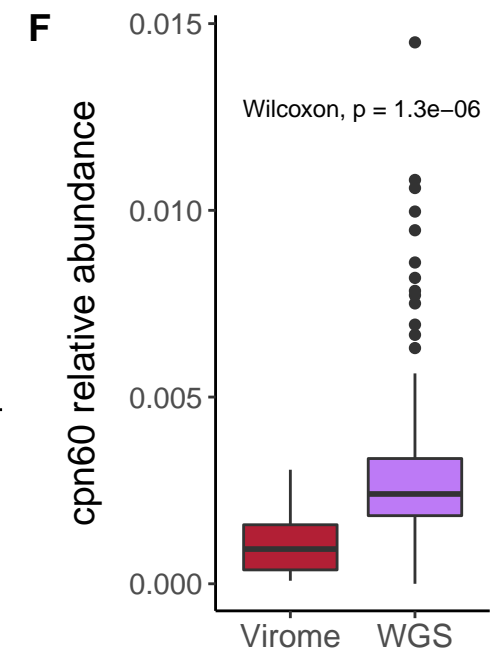

Supplement: Supplementary file 3 — Supplementary data [file 42003_2021_2666_MOESM3_ESM.zip › Supp_data_RatPD_wDarkMatter/Output_images/Fig1.pdf]

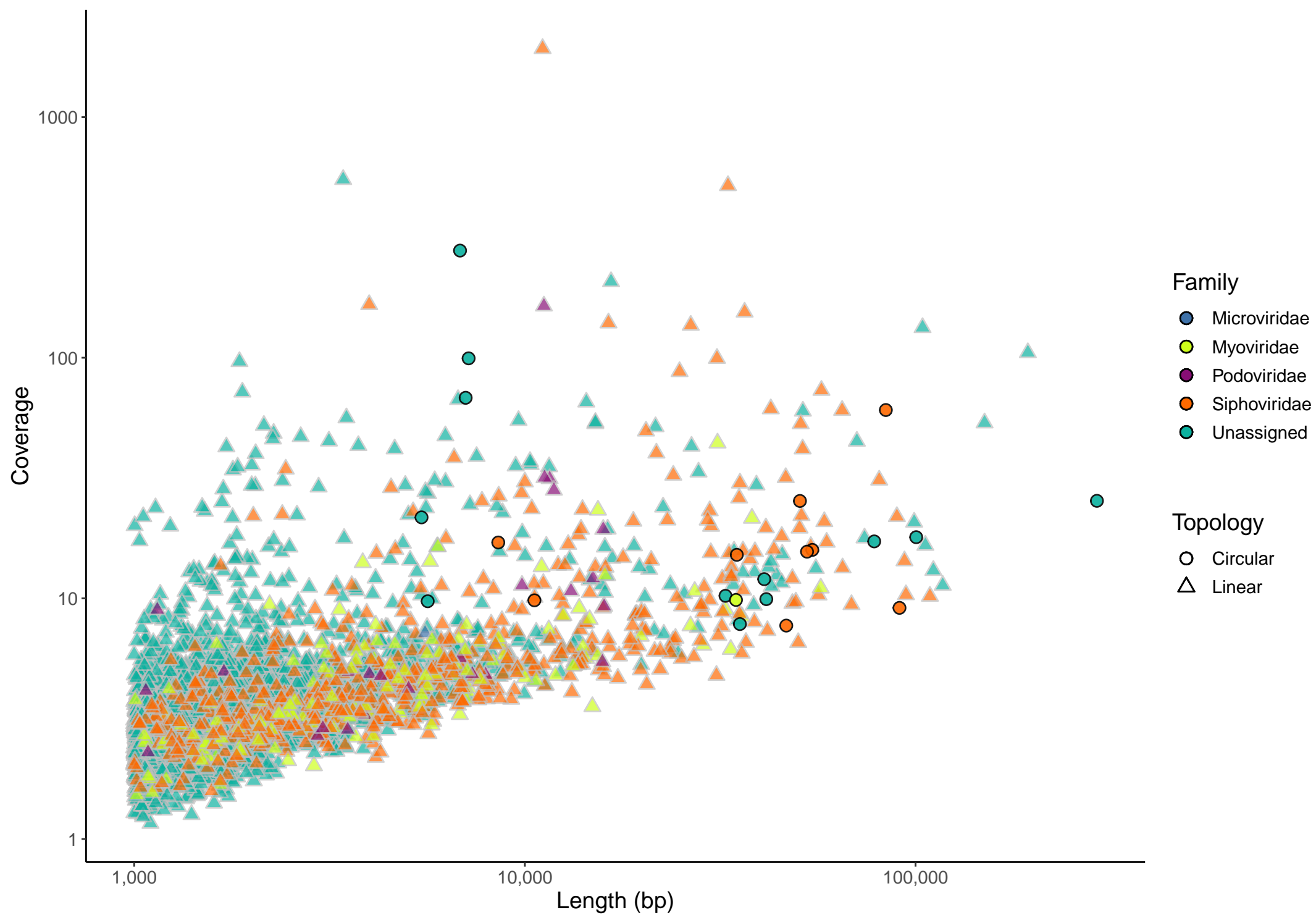

Supplement: Supplementary file 3 — Supplementary data [file 42003_2021_2666_MOESM3_ESM.zip › Supp_data_RatPD_wDarkMatter/Output_images/Fig1A.pdf]

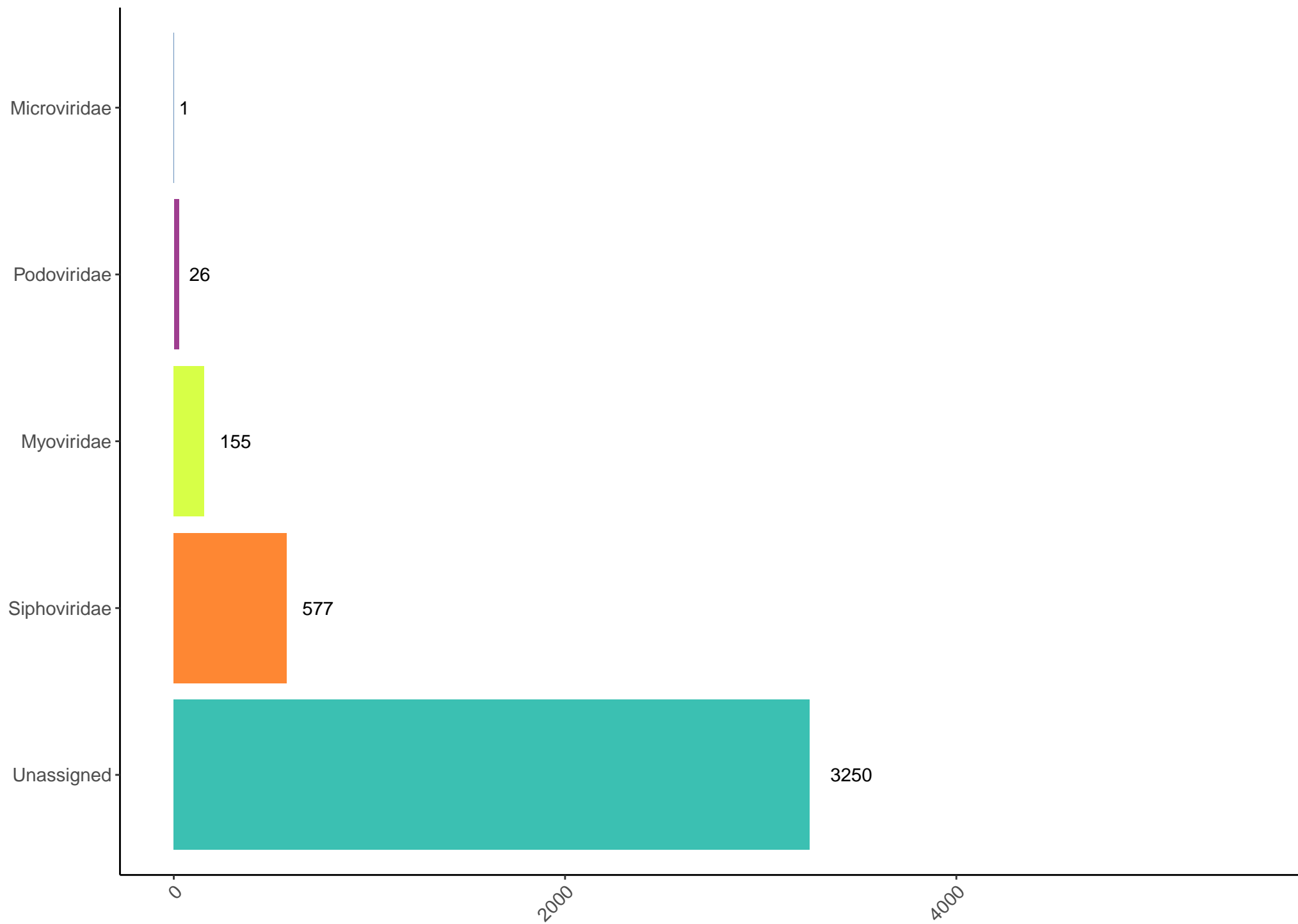

Supplement: Supplementary file 3 — Supplementary data [file 42003_2021_2666_MOESM3_ESM.zip › Supp_data_RatPD_wDarkMatter/Output_images/Fig1B.pdf]

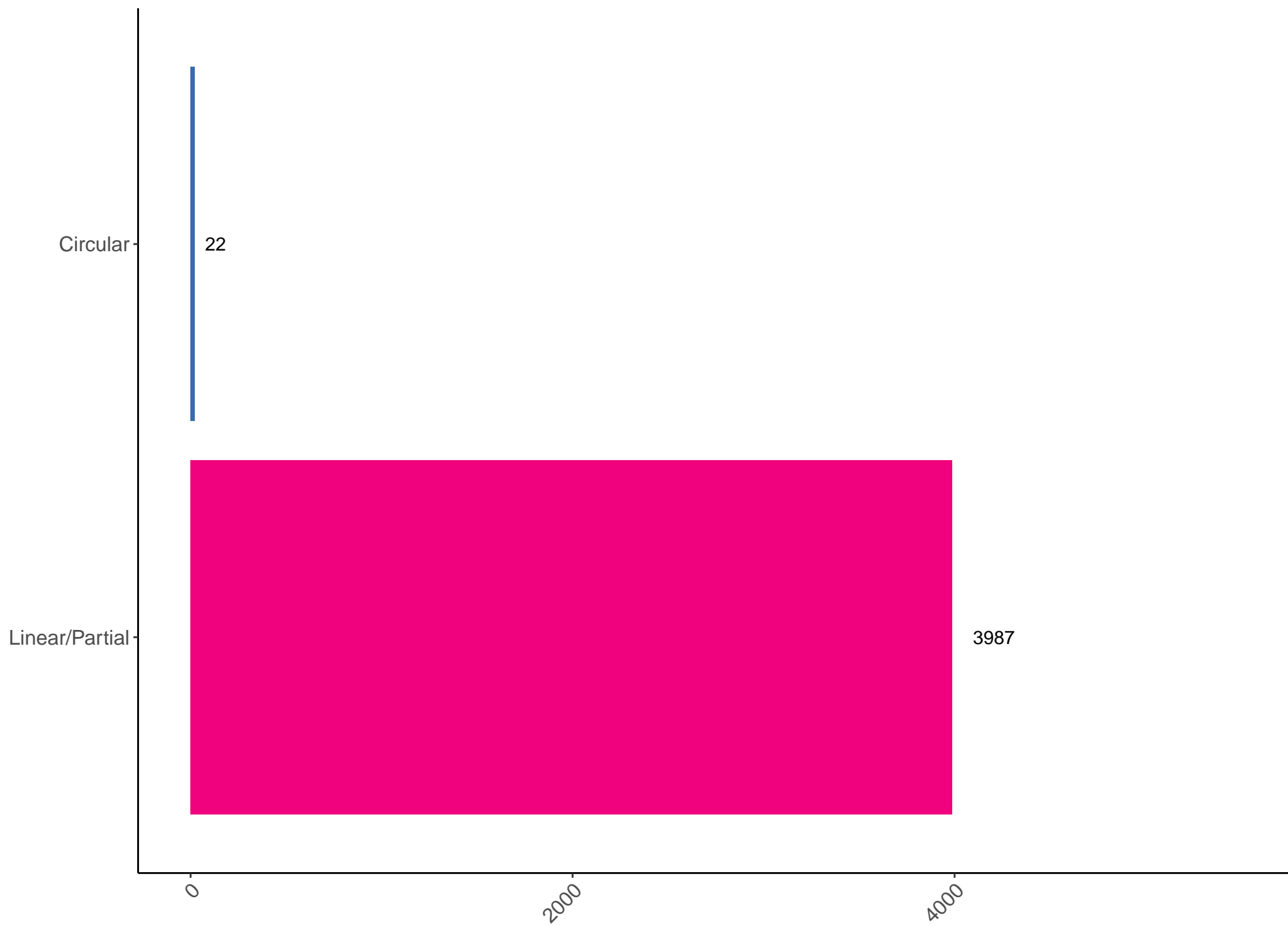

Supplement: Supplementary file 3 — Supplementary data [file 42003_2021_2666_MOESM3_ESM.zip › Supp_data_RatPD_wDarkMatter/Output_images/Fig1C.pdf]

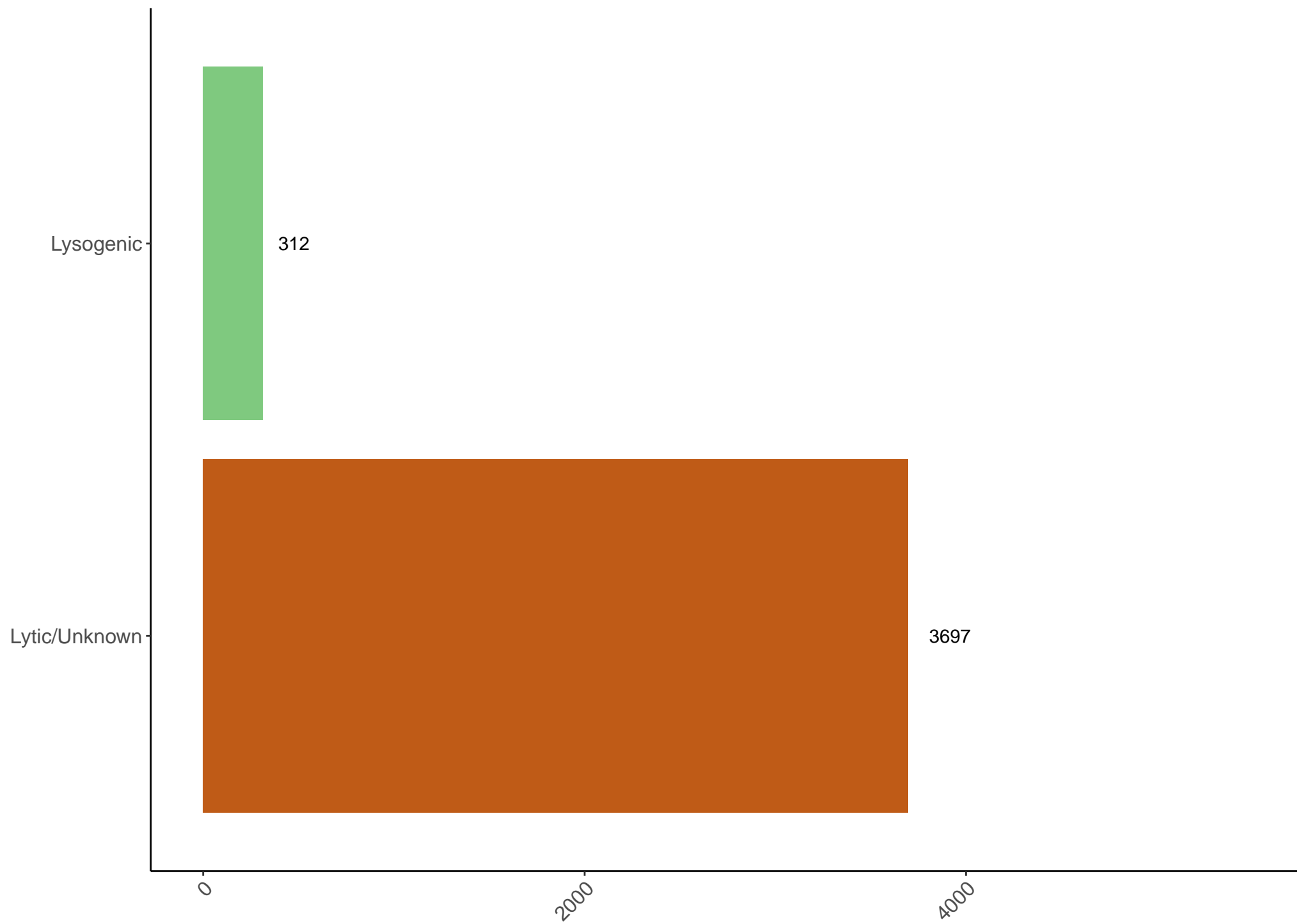

Supplement: Supplementary file 3 — Supplementary data [file 42003_2021_2666_MOESM3_ESM.zip › Supp_data_RatPD_wDarkMatter/Output_images/Fig1D.pdf]

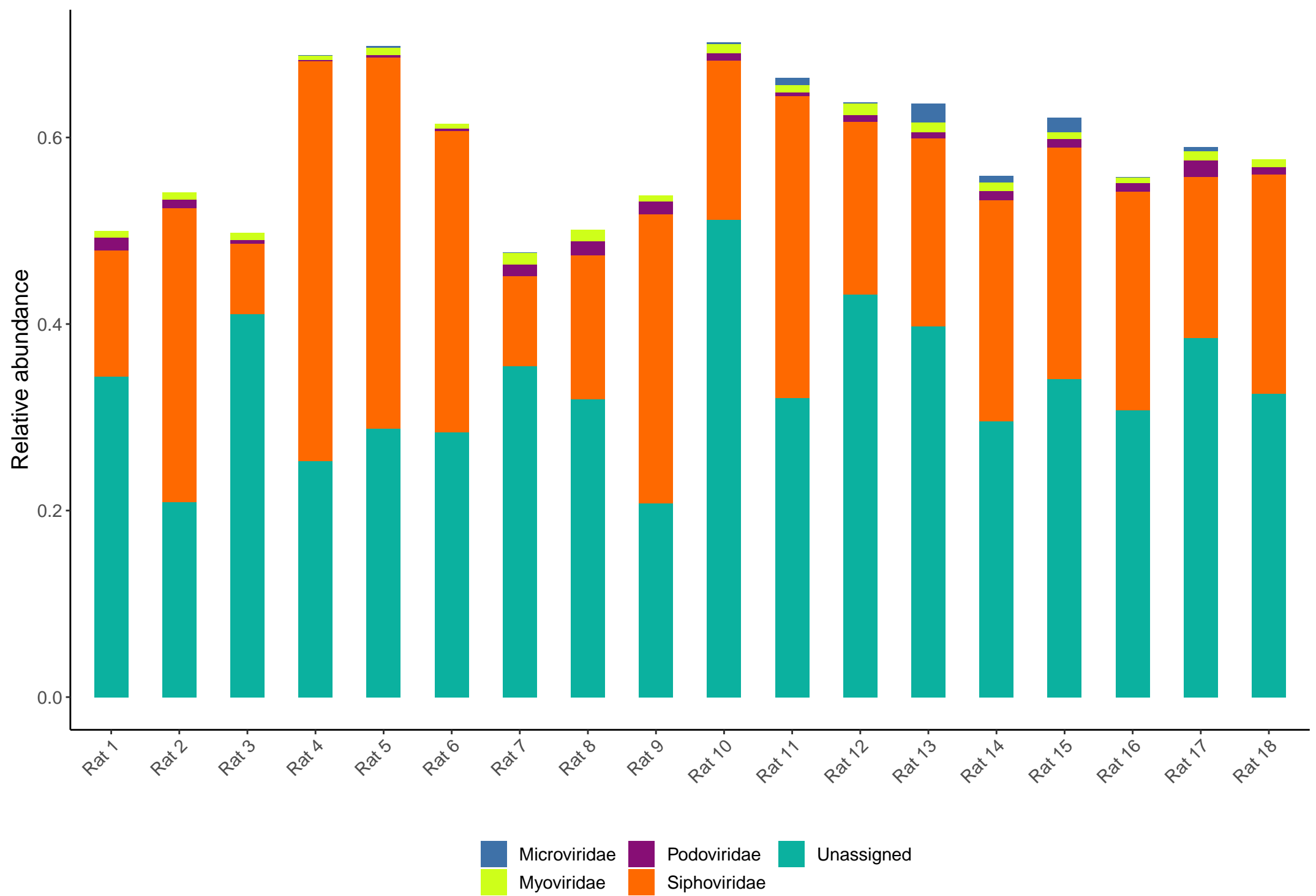

Supplement: Supplementary file 3 — Supplementary data [file 42003_2021_2666_MOESM3_ESM.zip › Supp_data_RatPD_wDarkMatter/Output_images/Fig1E.pdf]

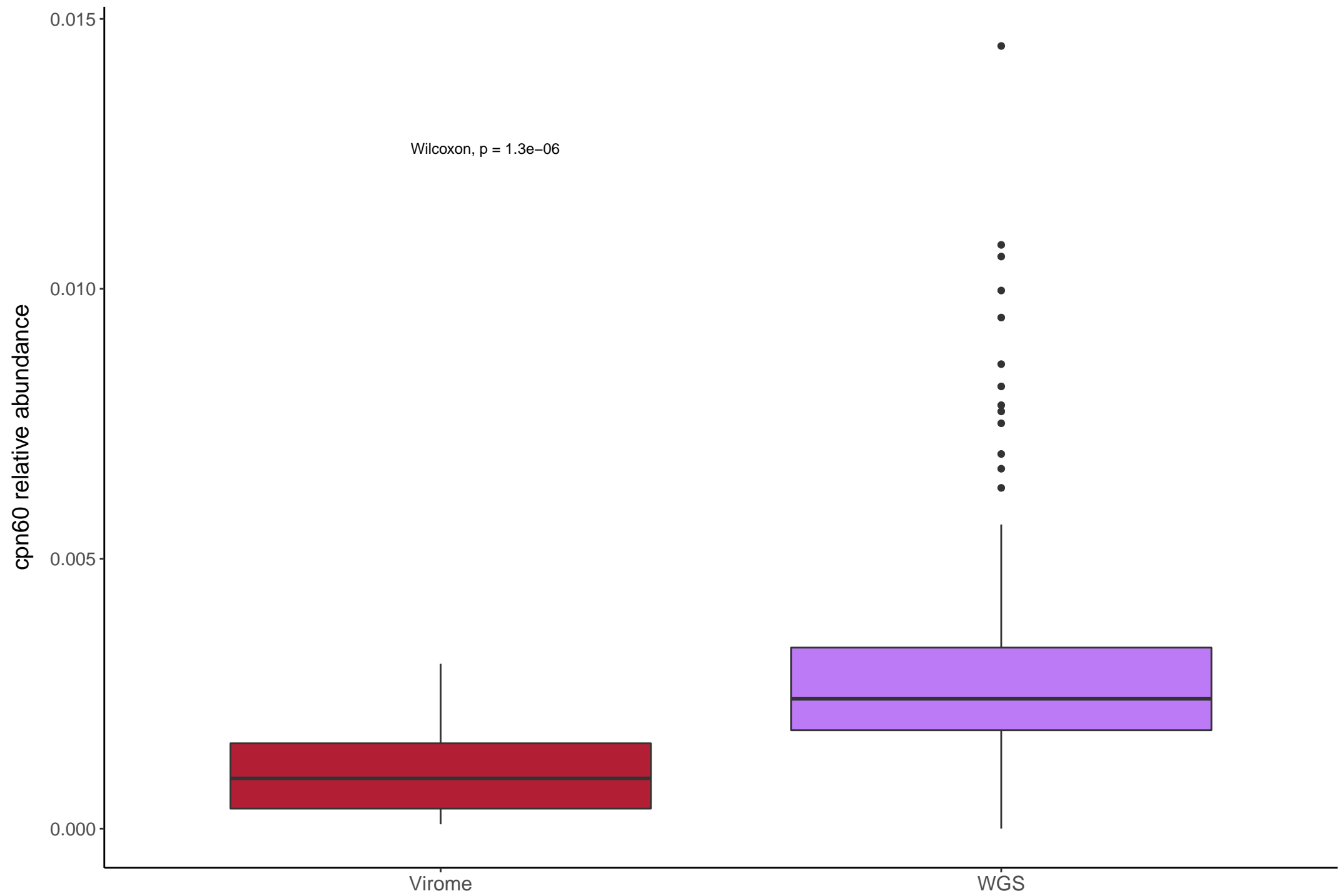

Supplement: Supplementary file 3 — Supplementary data [file 42003_2021_2666_MOESM3_ESM.zip › Supp_data_RatPD_wDarkMatter/Output_images/Fig1F.pdf]

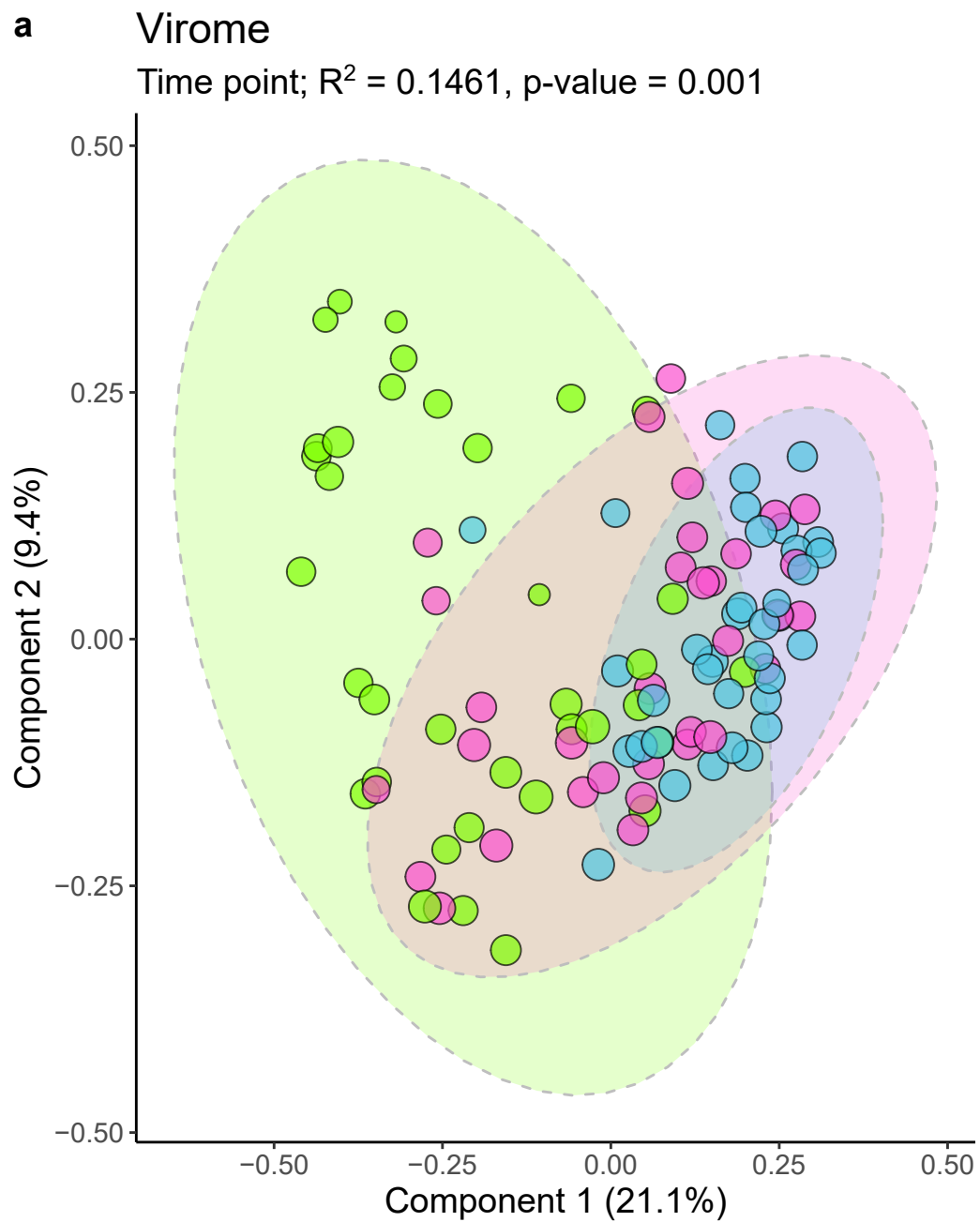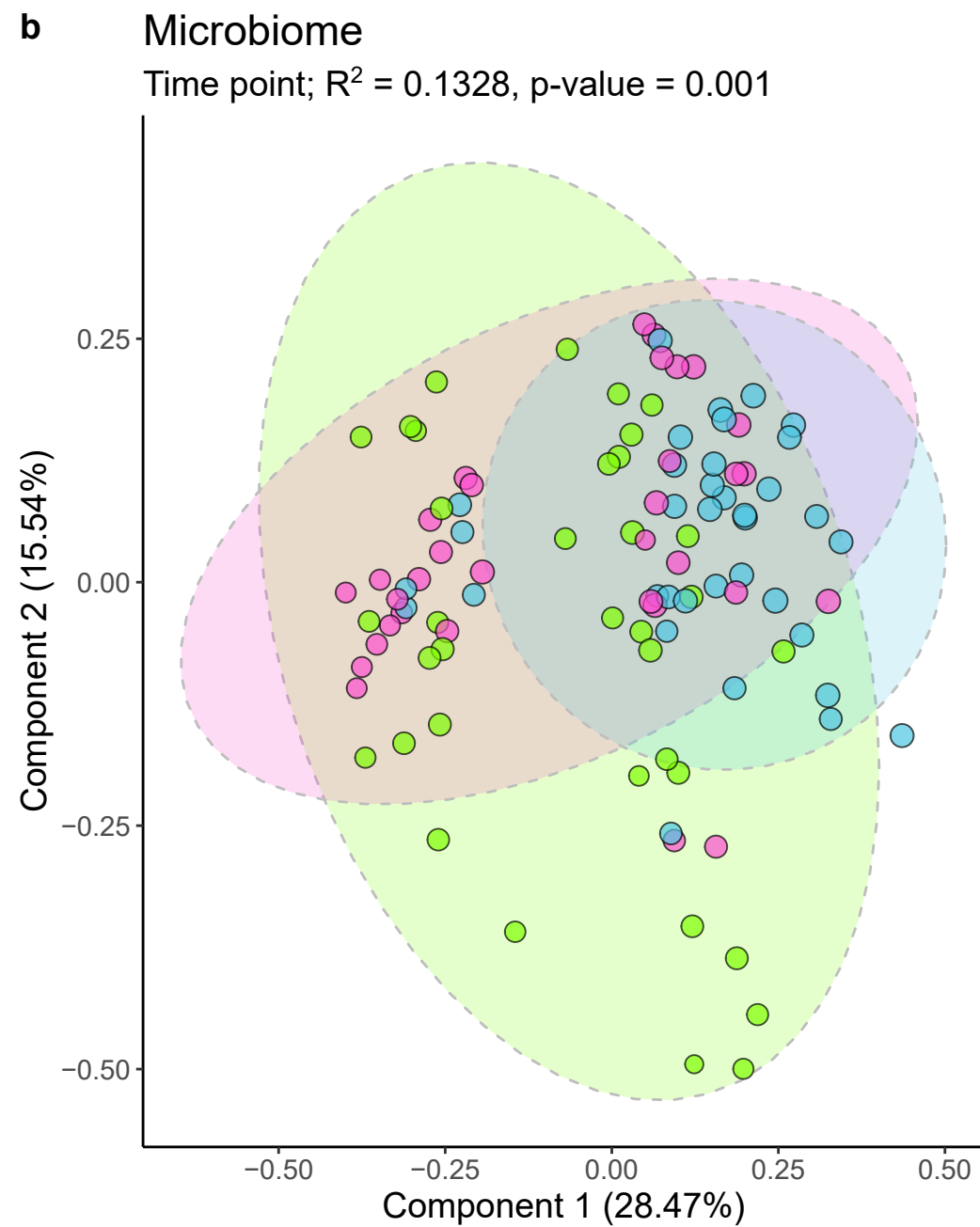

Shannon index ○ 1 ○ 2 ○ 3 ○ 4 ○ 5

Timepoint ● Base ● T1 ● T5

Supplement: Supplementary file 3 — Supplementary data [file 42003_2021_2666_MOESM3_ESM.zip › Supp_data_RatPD_wDarkMatter/Output_images/Fig2.pdf]

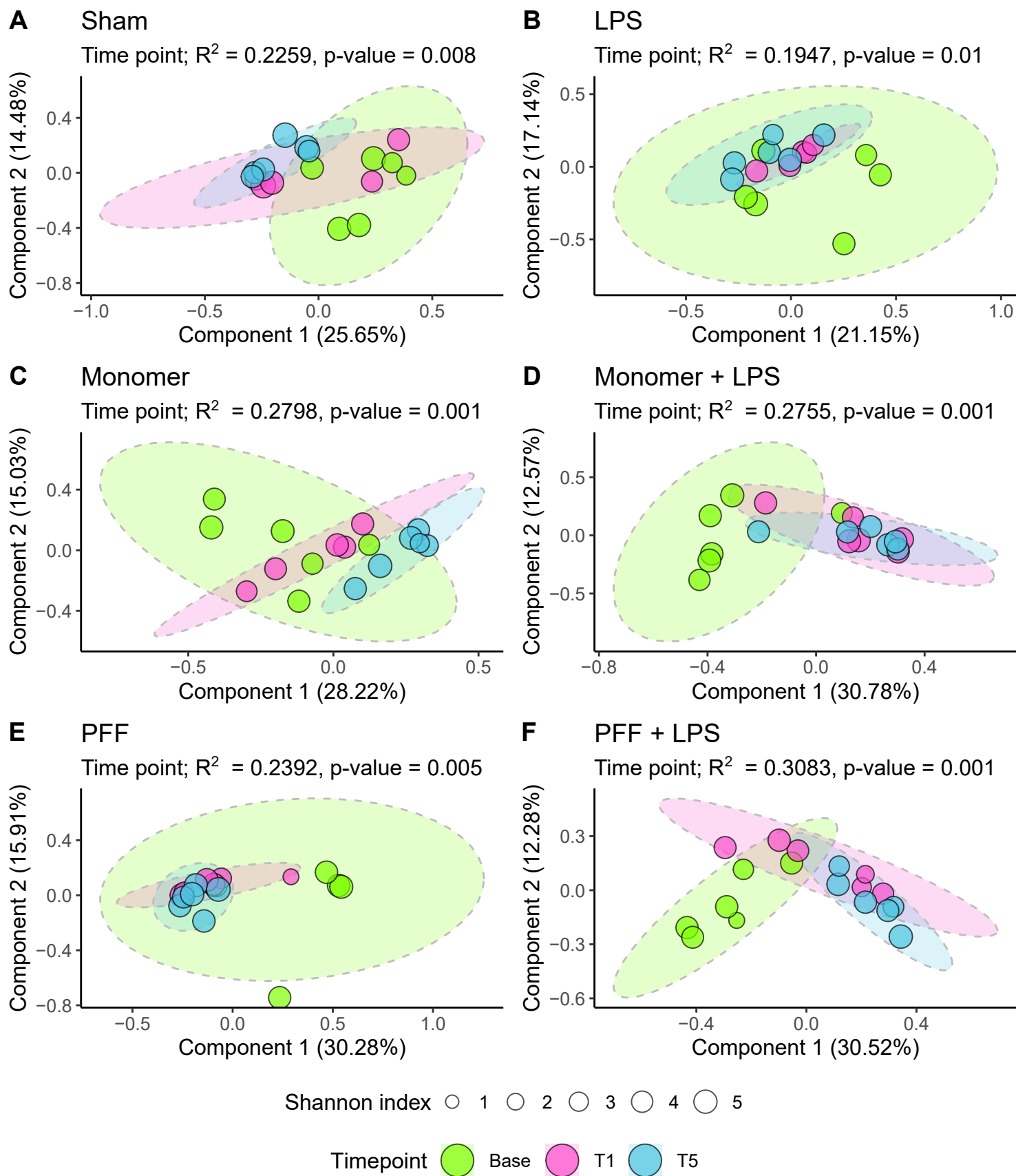

Supplement: Supplementary file 3 — Supplementary data [file 42003_2021_2666_MOESM3_ESM.zip › Supp_data_RatPD_wDarkMatter/Output_images/Fig3.pdf]

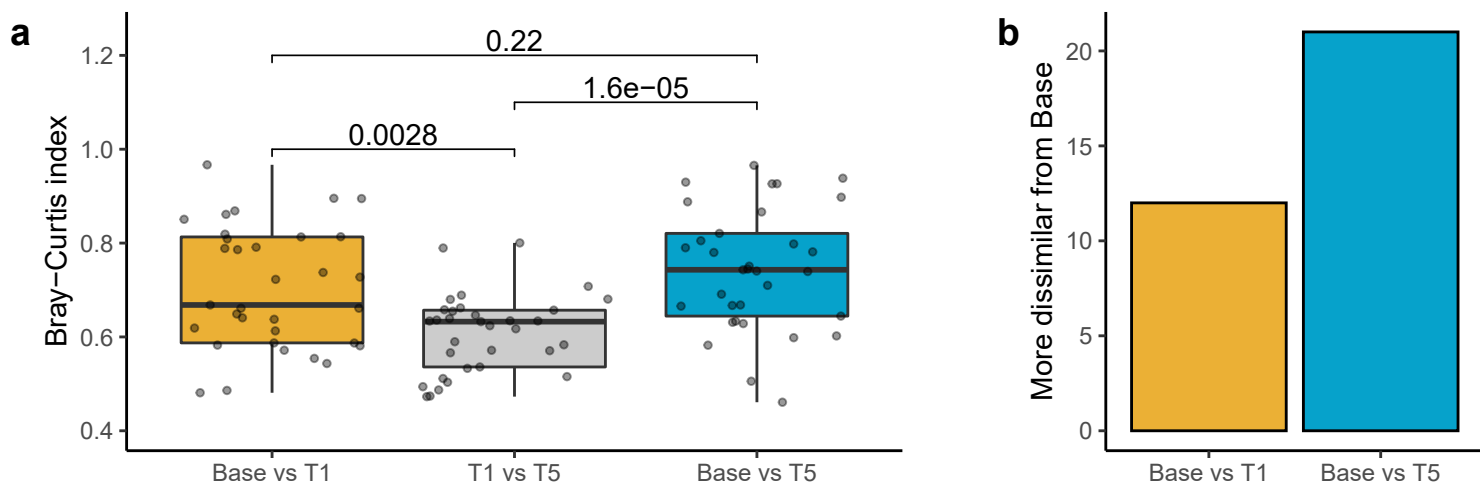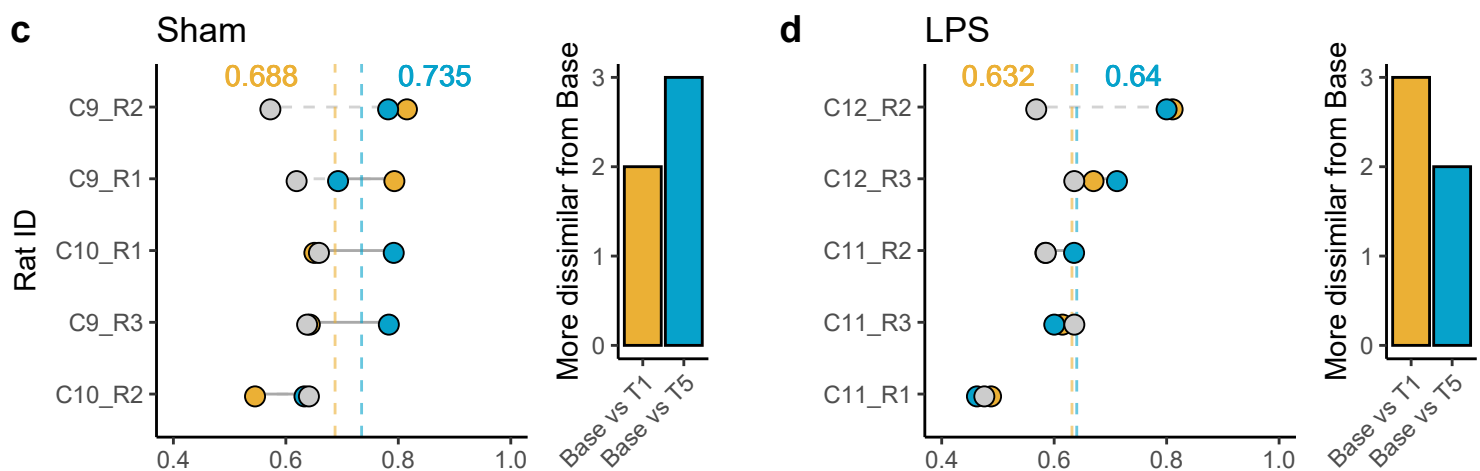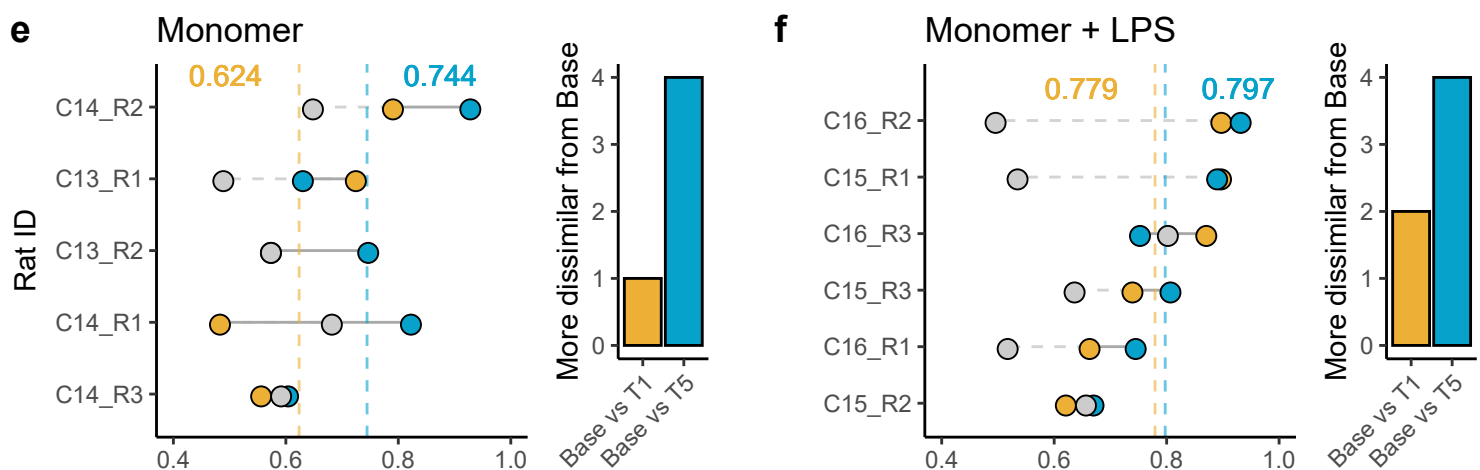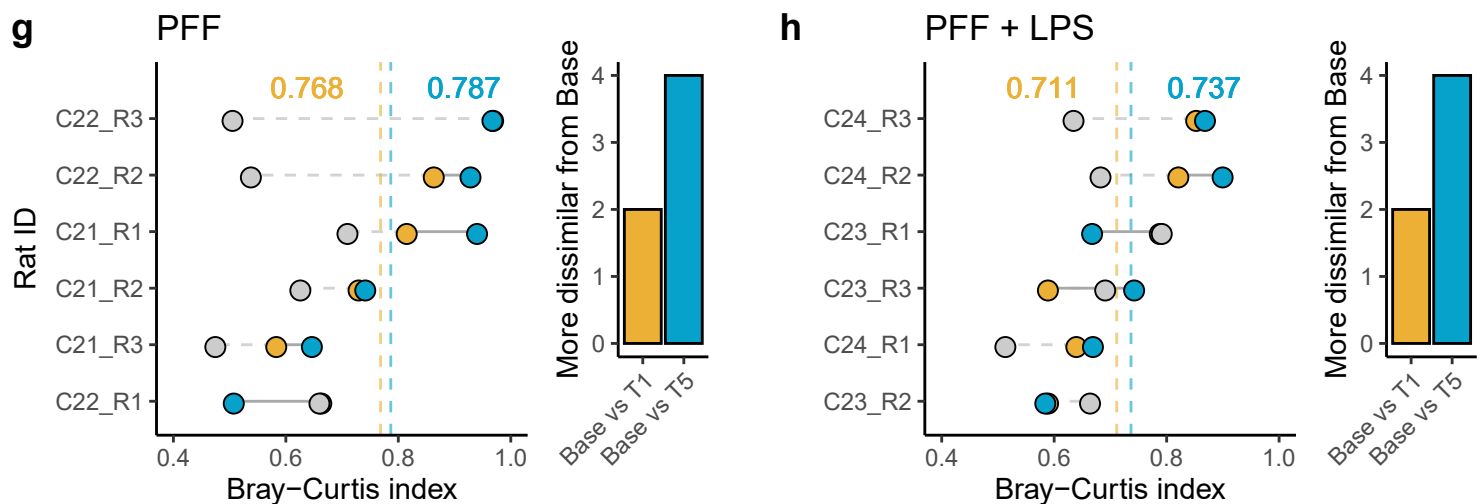

● Base vs T1

● T1 vs T5

● Base vs T5

Supplement: Supplementary file 3 — Supplementary data [file 42003_2021_2666_MOESM3_ESM.zip › Supp_data_RatPD_wDarkMatter/Output_images/Fig4.pdf]

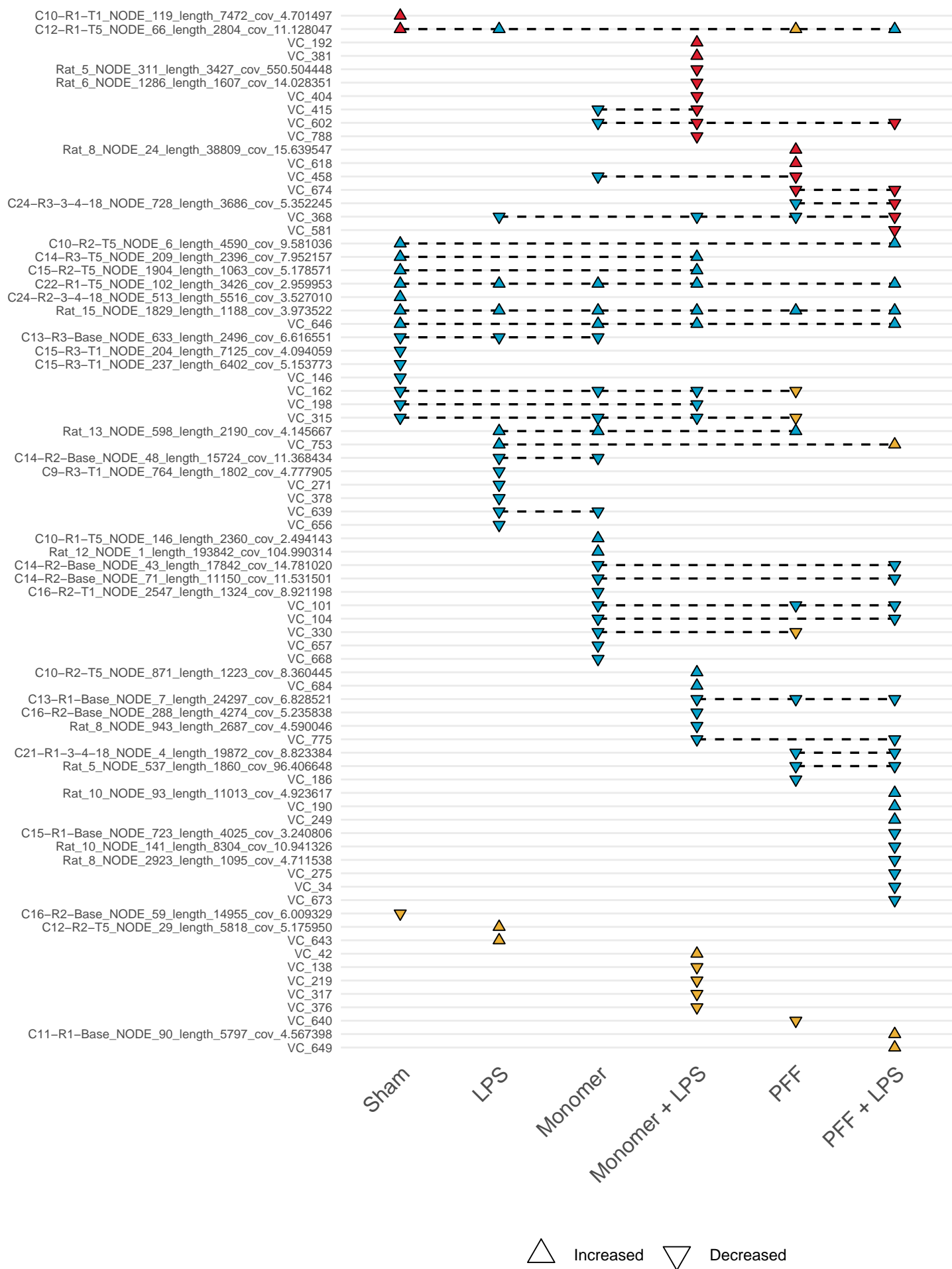

Significant in T1 and T5    Significant in T1    Significant in T5

Supplement: Supplementary file 3 — Supplementary data [file 42003_2021_2666_MOESM3_ESM.zip › Supp_data_RatPD_wDarkMatter/Output_images/Fig6.pdf]

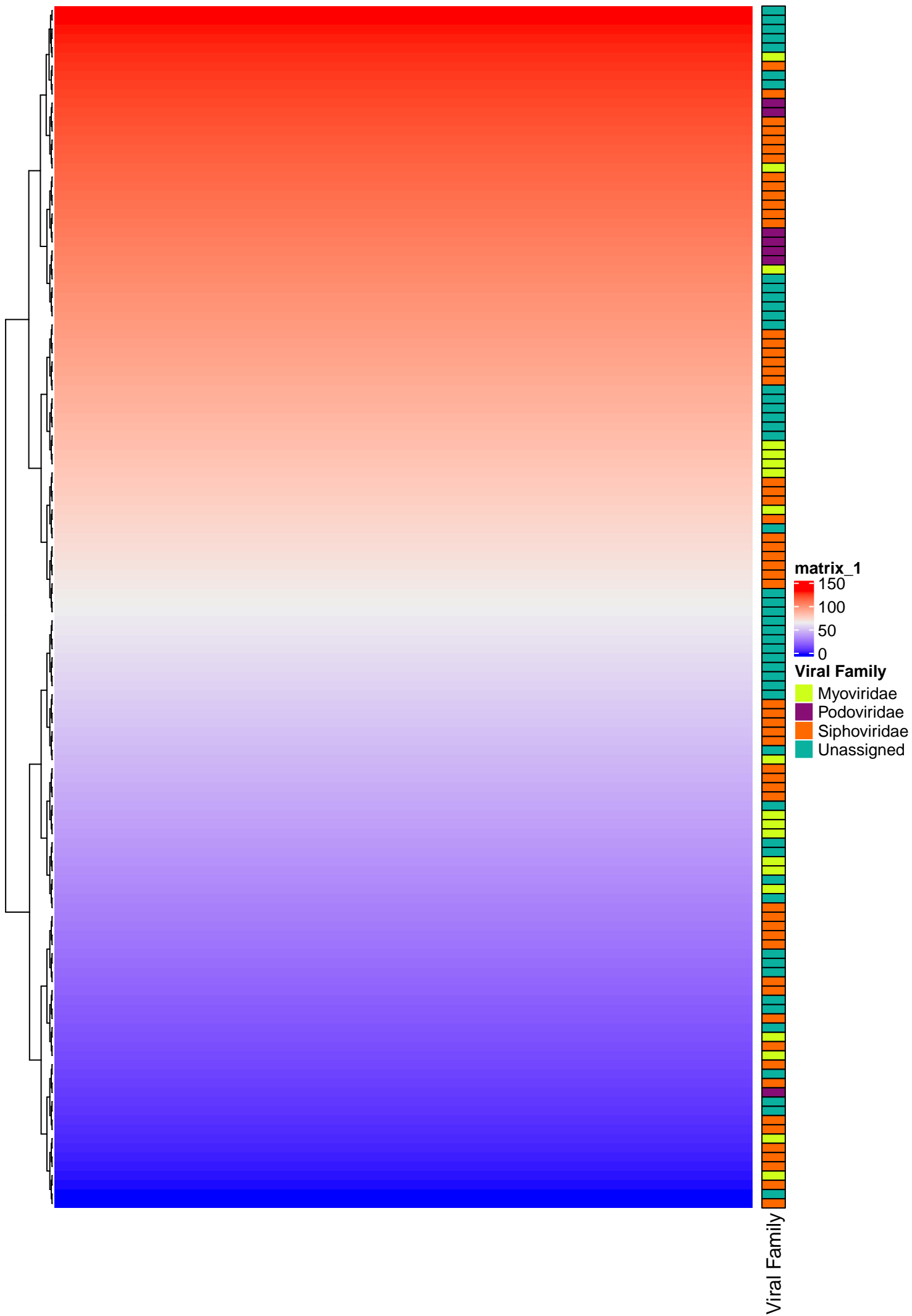

Supplement: Supplementary file 3 — Supplementary data [file 42003_2021_2666_MOESM3_ESM.zip › Supp_data_RatPD_wDarkMatter/Output_images/Fig6_annotationBar.pdf]

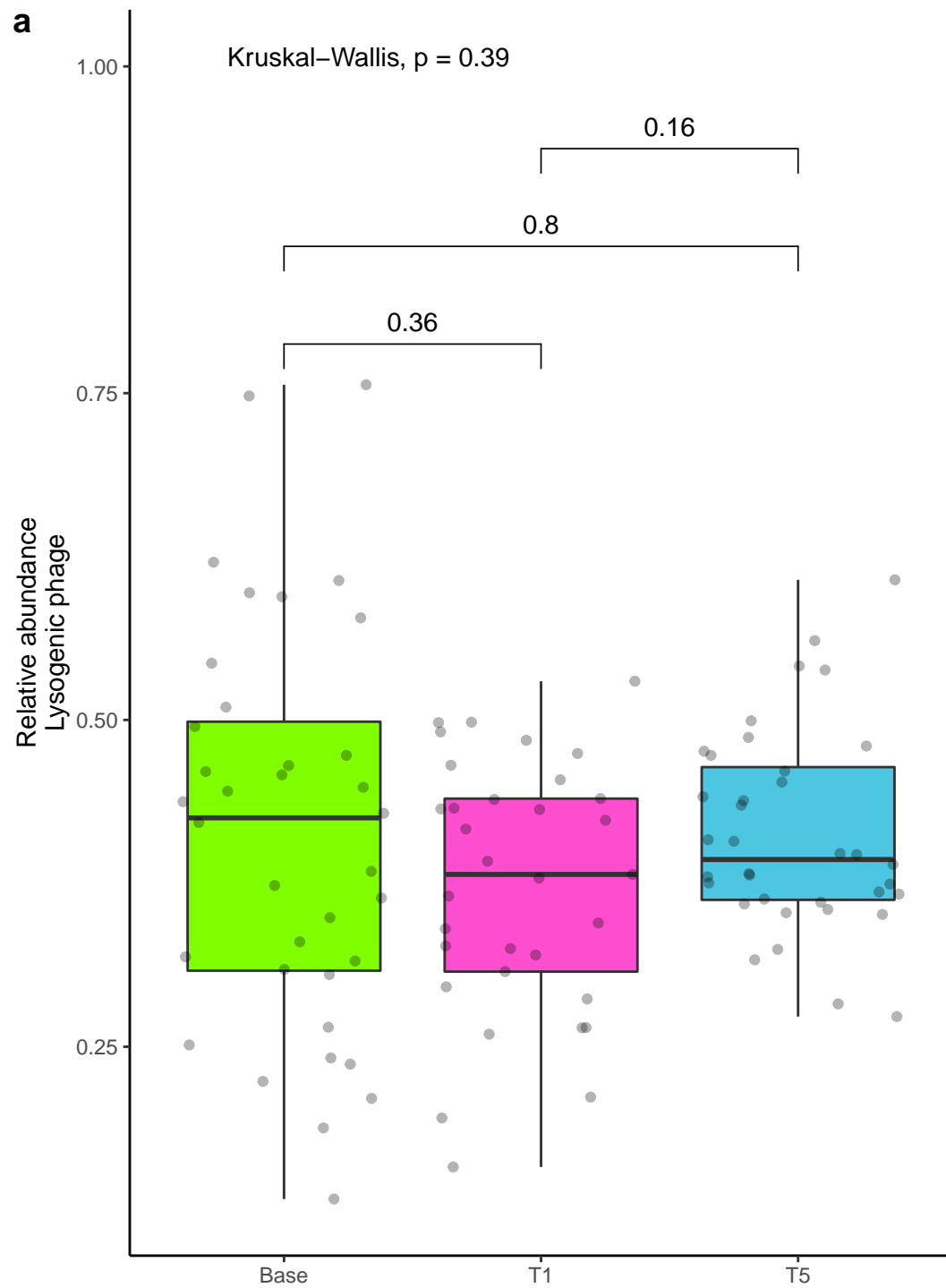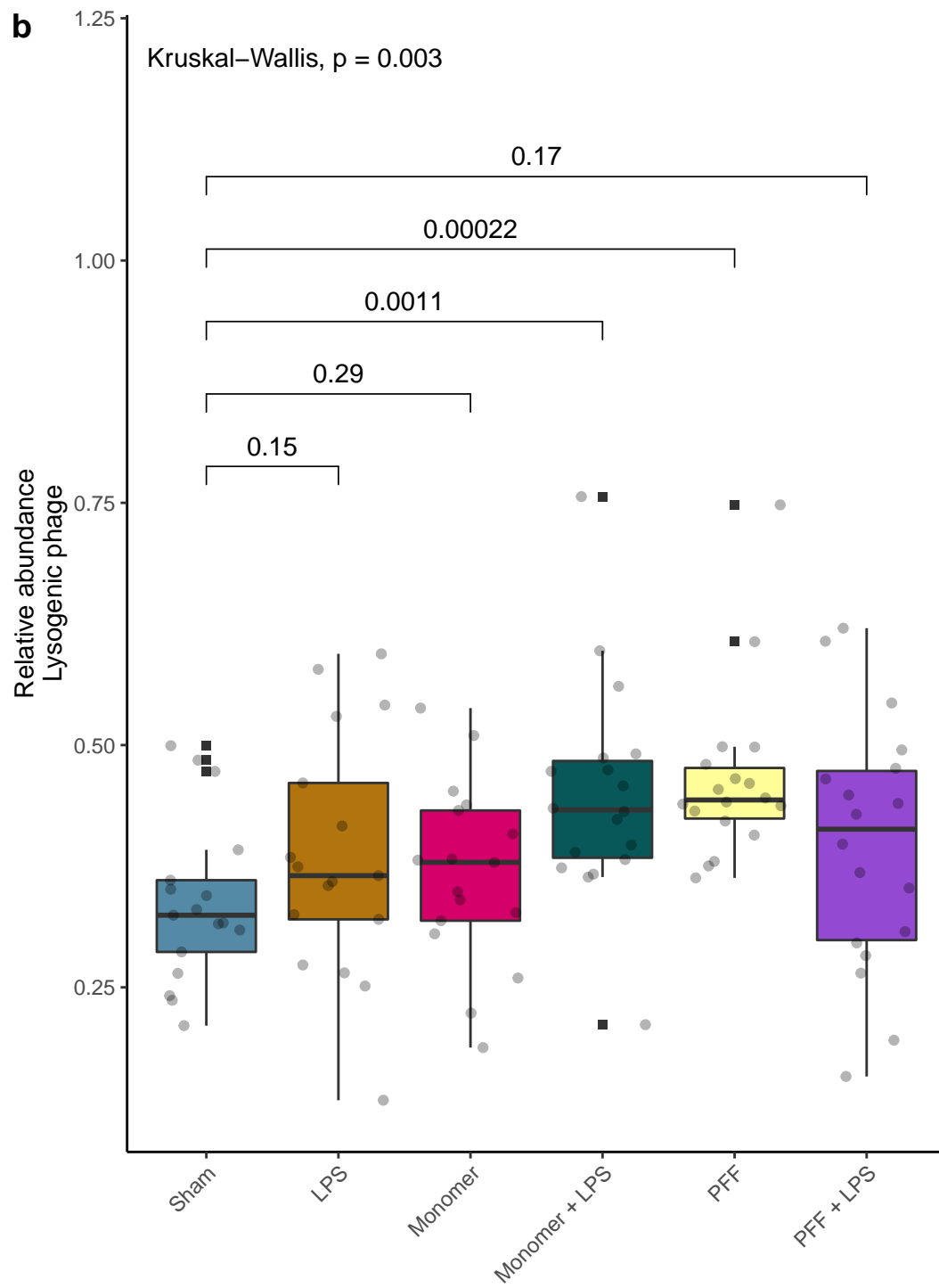

Supplement: Supplementary file 3 — Supplementary data [file 42003_2021_2666_MOESM3_ESM.zip › Supp_data_RatPD_wDarkMatter/Output_images/Supp_lyso_plots.pdf]

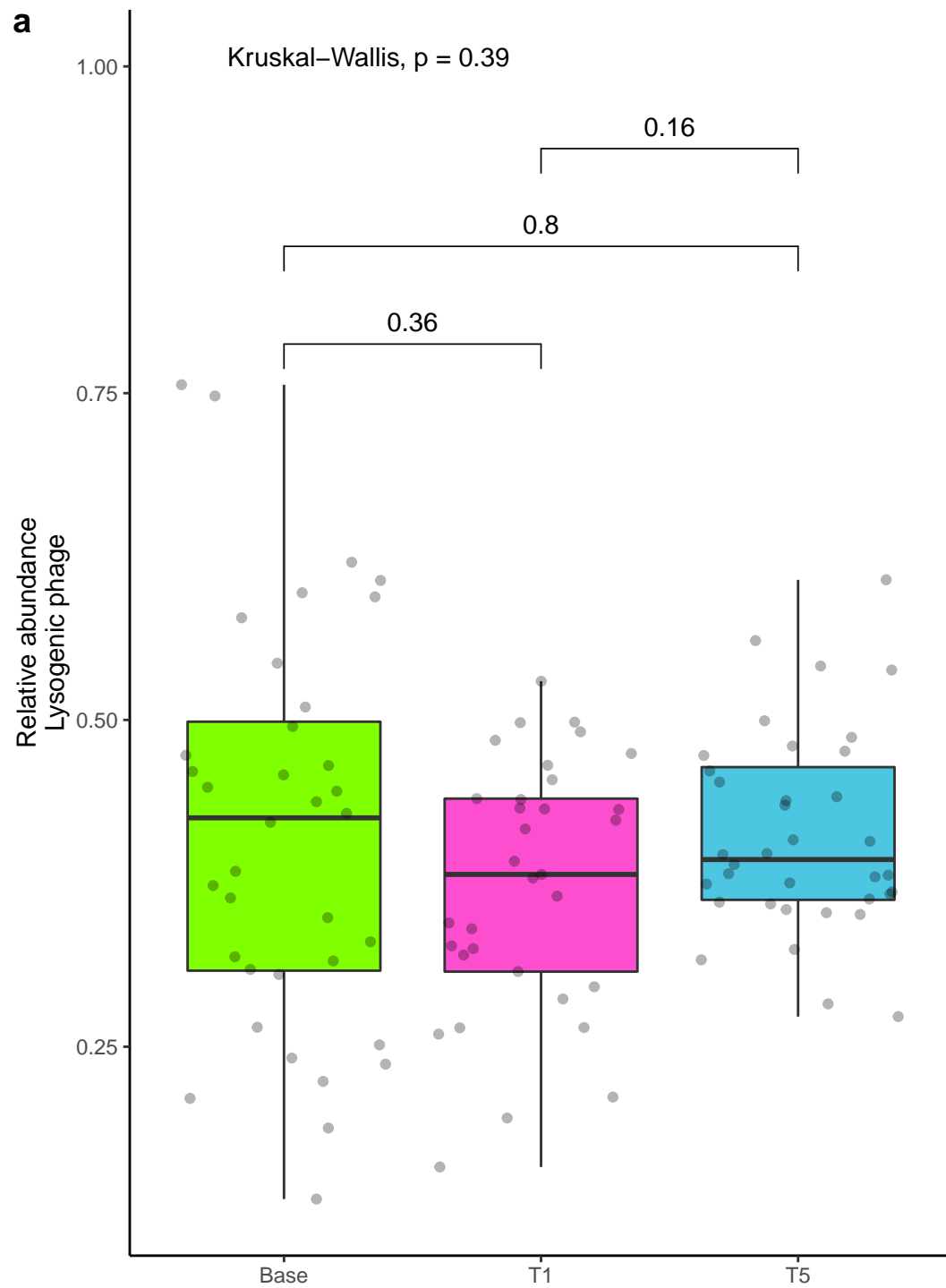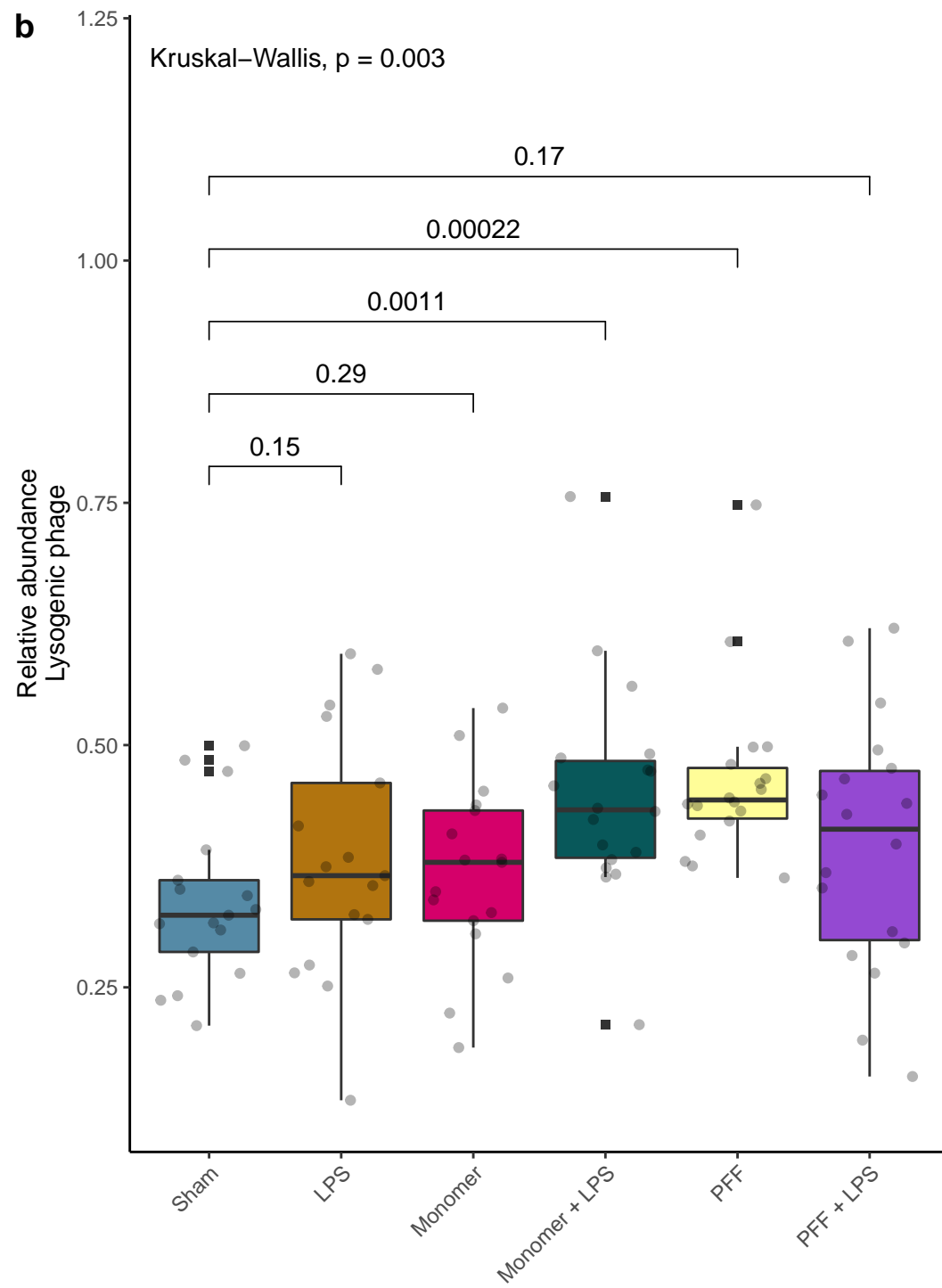

Supplement: Supplementary file 3 — Supplementary data [file 42003_2021_2666_MOESM3_ESM.zip › Supp_data_RatPD_wDarkMatter/Output_images/Supp_lyso_plots_SuppFig2.pdf]

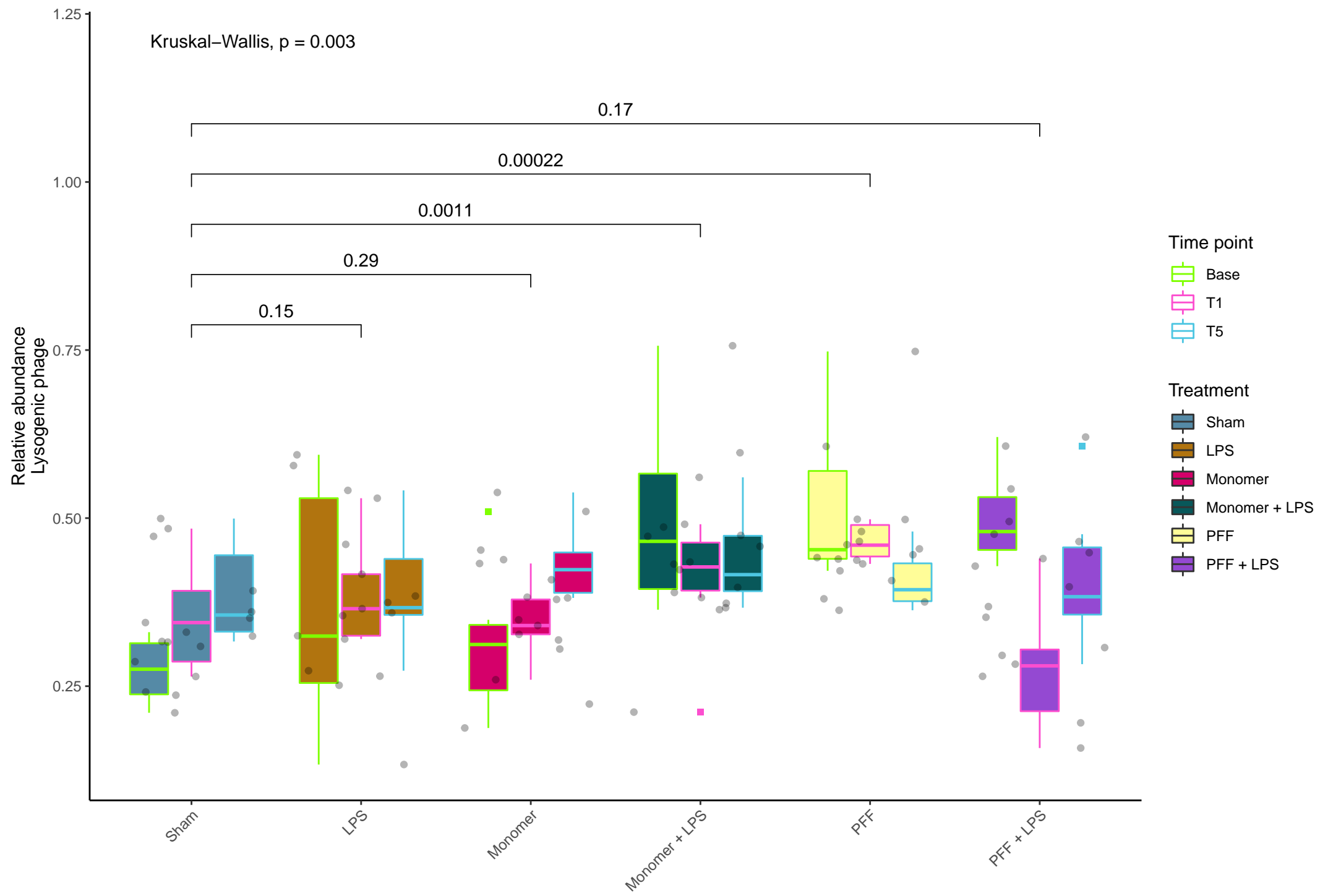

Supplement: Supplementary file 3 — Supplementary data [file 42003_2021_2666_MOESM3_ESM.zip › Supp_data_RatPD_wDarkMatter/Output_images/Supp_lysoTreatment_plots.pdf]

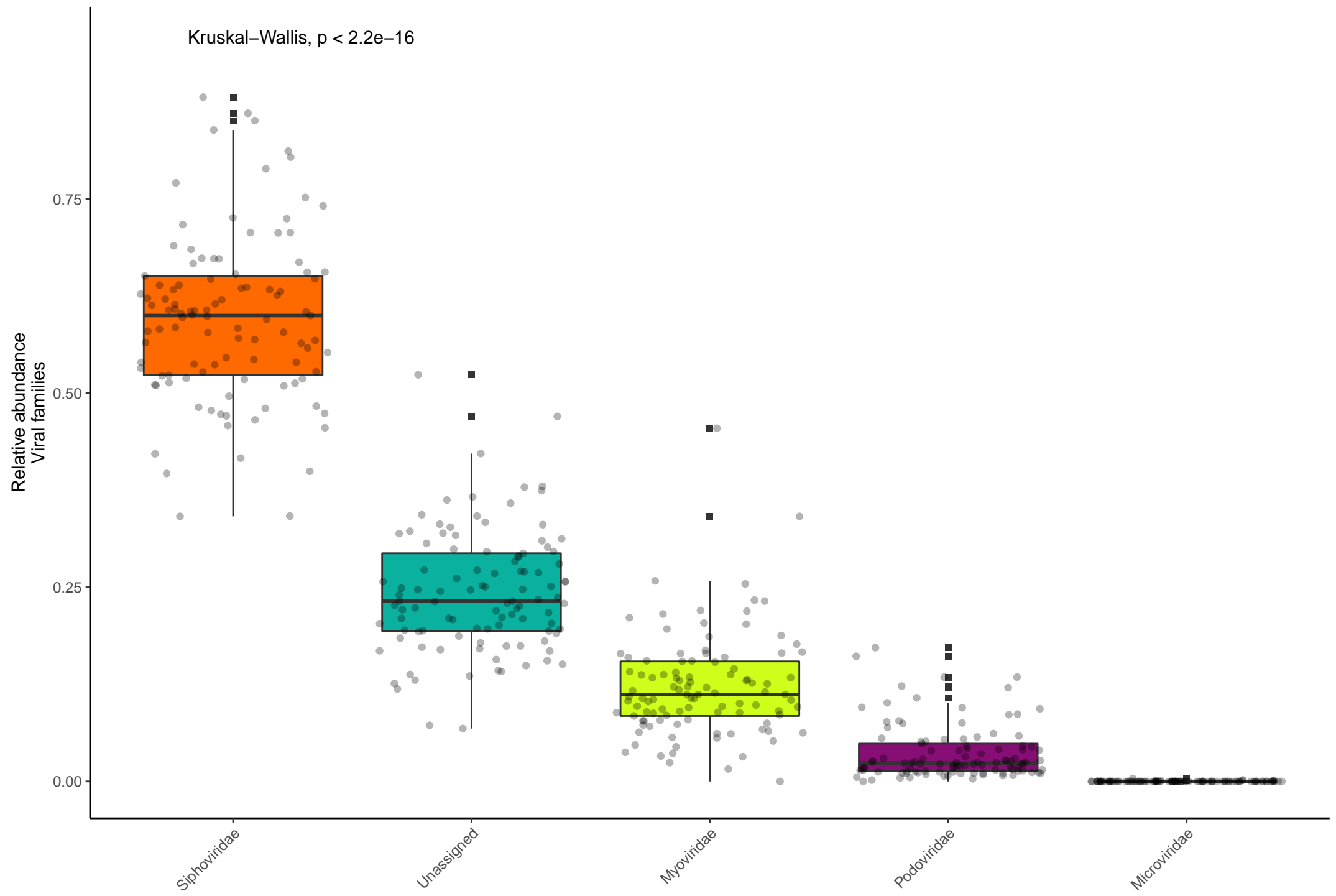

Supplement: Supplementary file 3 — Supplementary data [file 42003_2021_2666_MOESM3_ESM.zip › Supp_data_RatPD_wDarkMatter/Output_images/Supp_relAbund_WGStaxa.pdf]

PFF

PFF + LPS

Monomer

Monomer + LP

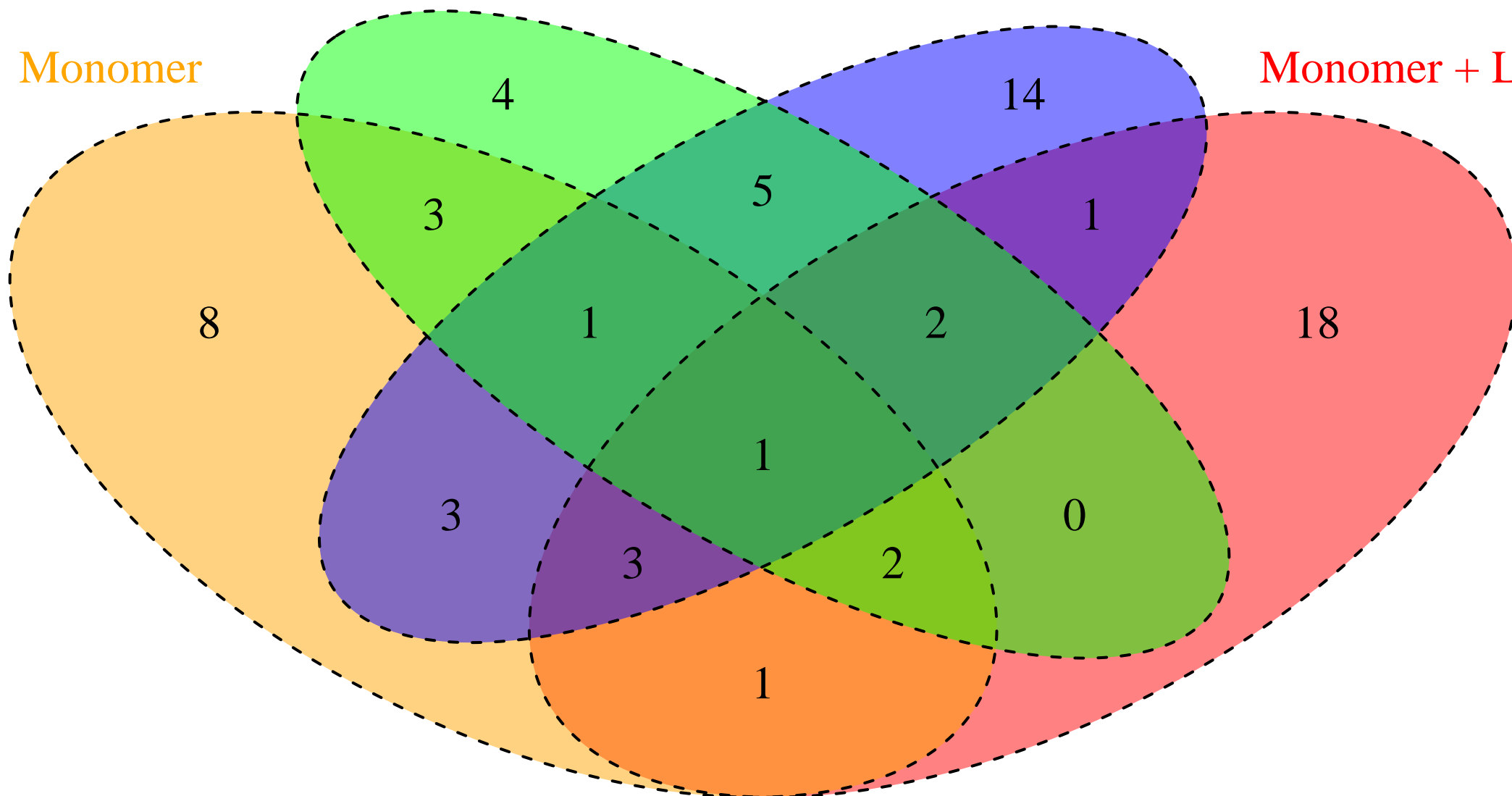

Supplement: Supplementary file 3 — Supplementary data [file 42003_2021_2666_MOESM3_ESM.zip › Supp_data_RatPD_wDarkMatter/Output_images/Supp_venn_overlap_SuppFig3.pdf]
