# Supplementary material for: Alpha-synuclein alters the faecal viromes of rats in a gut-initiated model of Parkinson’s disease
Source: Commun Biol. 2021 Sep 29;4:1140. doi: 10.1038/s42003-021-02666-1 (PMC8481466; doi:10.1038/s42003-021-02666-1)
Supplement: Supplementary file 4 — Reporting Summary [file 42003_2021_2666_MOESM4_ESM.pdf]

nature research

Corresponding author(s): Stephen Stockdale
Last updated by author(s): Aug 16, 2021

Reporting Summary

Nature Research wishes to improve the reproducibility of the work that we publish. This form provides structure for consistency and transparency in reporting. For further information on Nature Research policies, see our Editorial Policies and the Editorial Policy Checklist.

Statistics

For all statistical analyses, confirm that the following items are present in the figure legend, table legend, main text, or Methods section.

n/a	Confirmed	
☐	☒	The exact sample size (*n*) for each experimental group/condition, given as a discrete number and unit of measurement
☐	☒	A statement on whether measurements were taken from distinct samples or whether the same sample was measured repeatedly
☐	☒	The statistical test(s) used AND whether they are one- or two-sided<br>*Only common tests should be described solely by name; describe more complex techniques in the Methods section.*
☐	☒	A description of all covariates tested
☐	☒	A description of any assumptions or corrections, such as tests of normality and adjustment for multiple comparisons
☐	☒	A full description of the statistical parameters including central tendency (e.g. means) or other basic estimates (e.g. regression coefficient) AND variation (e.g. standard deviation) or associated estimates of uncertainty (e.g. confidence intervals)
☐	☒	For null hypothesis testing, the test statistic (e.g. F, t, r) with confidence intervals, effect sizes, degrees of freedom and P value noted<br>*Give P values as exact values whenever suitable.*
☒	☐	For Bayesian analysis, information on the choice of priors and Markov chain Monte Carlo settings
☒	☐	For hierarchical and complex designs, identification of the appropriate level for tests and full reporting of outcomes
☒	☐	Estimates of effect sizes (e.g. Cohen's d, Pearson's r), indicating how they were calculated

Our web collection on statistics for biologists contains articles on many of the points above.

Software and code

Policy information about availability of computer code

Data collection	Raw sequencing data was cleaned and assembled after sequencing. Freely-available, unmodified programmes were implemented and their use and references are detailed in the manuscript's methods section. Programmes for data collection and preparation include: FastQC, Trimmomatic, metaSPAdes, and Prodigal.
Data analysis	Viral sequencing data was analysed with reads mapped against a final viral database. Freely-available, unmodified programmes were implemented and their use and references are detailed in the manuscript's methods section. Programmes include: BLAST, VirSorter, HMMER, SAMTools, BEDTools, and Bowtie2. Additional analysis was conducted in R, with the custom code and packages required to repeat the analysis and images provided with the manuscript as Supplementary data.

For manuscripts utilizing custom algorithms or software that are central to the research but not yet described in published literature, software must be made available to editors and reviewers. We strongly encourage code deposition in a community repository (e.g. GitHub). See the Nature Research guidelines for submitting code & software for further information.

Data

Policy information about availability of data

All manuscripts must include a data availability statement. This statement should provide the following information, where applicable:

- Accession codes, unique identifiers, or web links for publicly available datasets
- A list of figures that have associated raw data
- A description of any restrictions on data availability

All sequencing data has been made publicly available before publication. Custom code is provided as supplementary data with the manuscript, and will also be maintained on an open access repository: http://doi.org/10.6084/m9.figshare.14332985. Data availability statements are included in the manuscript.

Field-specific reporting

Please select the one below that is the best fit for your research. If you are not sure, read the appropriate sections before making your selection.

☒ Life sciences ☐ Behavioural & social sciences ☐ Ecological, evolutionary & environmental sciences

For a reference copy of the document with all sections, see nature.com/documents/nr-reporting-summary-flat.pdf

Life sciences study design

All studies must disclose on these points even when the disclosure is negative.

Sample size	Sample sizes were a priori powered and checked by an in-house statistician before ethical review and project authorization was granted.
Data exclusions	No samples were excluded from analyses.
Replication	Due to the nature of the study, reproduction was not performed. The manuscript does outline an analysis of cage effects to ensure there were no significant outliers.
Randomization	All rats were randomised into cages and treatments.
Blinding	Separate authors led the animal experiments, sequencing preparation, and analysis. An uninformative code was assigned to faecal samples during processing - which was decrypted during final analysis. Therefore, only the researcher performing the sequencing preparation was totally naive to the treatment groups. The specialist nature of the surgical preparation and injection of alpha-synuclein into the mesenteric plexus required the author leading the animal studies to know the treatments received by each animal.

Reporting for specific materials, systems and methods

We require information from authors about some types of materials, experimental systems and methods used in many studies. Here, indicate whether each material, system or method listed is relevant to your study. If you are not sure if a list item applies to your research, read the appropriate section before selecting a response.

Materials & experimental systems

n/a	Involved in the study	
☒	☐	Antibodies
☒	☐	Eukaryotic cell lines
☒	☐	Palaeontology and archaeology
☐	☒	Animals and other organisms
☒	☐	Human research participants
☒	☐	Clinical data
☒	☐	Dual use research of concern

Methods

n/a	Involved in the study	
☒	☐	ChIP-seq
☒	☐	Flow cytometry
☒	☐	MRI-based neuroimaging

Animals and other organisms

Policy information about studies involving animals; ARRIVE guidelines recommended for reporting animal research

Laboratory animals	Adult male Sprague-Dawley rats were purchased from Envigo, UK.
Wild animals	NA
Field-collected samples	NA
Ethics oversight	Health Products Regulatory Authority, Ireland under project authorisation AE19130/P036.

Note that full information on the approval of the study protocol must also be provided in the manuscript.
